# The loss of taste genes in cetaceans

**DOI:** 10.1186/s12862-014-0218-8

**Published:** 2014-10-12

**Authors:** Kangli Zhu, Xuming Zhou, Shixia Xu, Di Sun, Wenhua Ren, Kaiya Zhou, Guang Yang

**Affiliations:** Jiangsu Key Laboratory for Biodiversity and Biotechnology, College of Life Sciences, Nanjing Normal University, Nanjing, 210023 China; Division of Genetics, Department of Medicine, Brigham and Women’s Hospital, Harvard Medical School, Boston, MA 02115 USA

**Keywords:** Cetacean, Taste genes, Pseudogenization, Molecular evolution

## Abstract

**Background:**

Five basic taste modalities, sour, sweet, bitter, salt and umami, can be distinguished by humans and are fundamental for physical and ecological adaptations in mammals. Molecular genetic studies of the receptor genes for these tastes have been conducted in terrestrial mammals; however, little is known about the evolution and adaptation of these genes in marine mammals.

**Results:**

Here, all five basic taste modalities, sour, sweet, bitter, salt and umami, were investigated in cetaceans. The sequence characteristics and evolutionary analyses of taste receptor genes suggested that nearly all cetaceans may have lost all taste modalities except for that of salt.

**Conclusions:**

This is the first study to comprehensively examine the five basic taste modalities in cetaceans with extensive taxa sampling. Our results suggest that cetaceans have lost four of the basic taste modalities including sour, sweet, umami, and most of the ability to sense bitter tastes. The integrity of the candidate salt taste receptor genes in all the cetaceans examined may be because of their function in Na^+^ reabsorption, which is key to osmoregulation and aquatic adaptation.

**Electronic supplementary material:**

The online version of this article (doi:10.1186/s12862-014-0218-8) contains supplementary material, which is available to authorized users.

## Background

Cetaceans, commonly known as whales, dolphins and porpoises, have a mysterious history of transition from land to water. Numerous phylogenetic studies based on morphological as well as molecular characteristics have suggested that Cetacea is an independent clade nested within the mammalian order Artiodactyla (reviewed in [1]). Molecular studies have dated modern cetaceans (including toothed and baleen whales) to have originated about 34 Mya (Million years ago) [2,3]. Subsequently, cetaceans developed a series of adaptations to fully aquatic environments (e.g., loss of limbs, shortening of the skull, loss of sebaceous glands, echolocation ability in Odontoceti, and baleen plate in Mysticeti) [1,4,5]. However, the genetic basis for the origin and adaptation of this group of species is far from clear. Several studies have shown that many loci in cetaceans have gone through adaptive evolution, suggesting that some cetacean organs evolved adaptively while others degenerated. For example, the membrane motor protein gene *prestin*, which is associated with high-frequency hearing in vertebrates, was shown to undergo positive selection in echolocating dolphins [6-8], whereas the number of olfactory receptor family pseudogenes is significantly higher in cetaceans than in other mammals [9-12].

Five basic taste modalities, sour, sweet, bitter, salt, and umami, can be distinguished by humans and are fundamental for physical and ecological adaptations in mammals [13,14]. Among them, umami and sweet tastes are attractive and beneficial to animals’ ingestion of protein-rich and nutritious food. Salt at low concentrations is an attractive taste and is associated with Na^+^ reabsorption [15-18]. Bitter tastes can cause taste aversion, thus protecting mammals from ingesting toxic substances [19,20]. Sour tastes are unpleasant and can prevent the ingestion of unripe and decayed food resources [21]. The receptor genes of each taste modality have been identified in mammals. In particular, umami/sweet tastants are perceived by Tas1rs (taste receptor, type 1 receptors) belonging to the G-protein coupled receptor C subtype family. Tas1r1 or Tas1r2 are co-expressed with Tas1r3 to perceive umami or sweet tastants, respectively [22-27]. Bitter substances are perceived by Tas2rs (taste receptor, type 2 receptors) [28-30]. Chandrashekar et al. (2000) [29] demonstrated that a mouse T2R (mT2R-5) responded to the bitter tastant cycloheximide, and a human receptor hT2R-4 and a mouse receptor mT2R-8 responded to denatonium and 6-n-propyl-2-thiouracil. Jiang et al. (2012) [31] identified only 10 *Tas2rs* genes in dolphin genome and all these genes were proved to be pseudogenes. Thus, we used these 10 *Tas2rs* from cow and dog reference genomes to search the Yangtze River dolphin (or baiji, *Lipotes vexillifer*) genome for the raw members of these genes and further compared them to dolphin assembly. Eventually, we identified 8 *Tas2rs* excluding *Tas2r38* and *Tas2r62b* in baiji genome, so we used the 10 Tas2rs in Jiang et al. 2012 to conduct our experiment. Sour and salt taste receptors are ion channels. To date, several candidate sour taste receptors have been reported, including acid sensing ion channels (ASICs) [32], hyperpolarization activated cyclic nucleotide gated potassium channels (HCNs) [33], potassium channels [34], and polycystic kidney disease 1 L3 and 2 L1 heteromers (PKD1L3+ PKD2L1) [35-37]. Here we chose PKD2L1 to investigate whether cetaceans retained the sour taste modality, because mice lacking the *Pkd2l1* gene have reduced sour taste ability and some people who are sour-ageusic also showed loss of the *Pkd2l1* gene [38,39]. Opposite taste responses are observed for saline solutions of different concentrations; low concentrations are perceived as attractive while concentrated solutions are aversive. These opposing responses are reported to be perceived by different receptors and different pathways [14-16]. The epithelial sodium channel ENaC is involved in attractive sodium sensing and knockout of ENaCα in mice resulted in a complete loss of salt attraction and salt response [40,41].

Promoted by the discovery of taste receptor genes, the evolutionary history of taste perception under certain ecological and feeding behaviors has been studied in detail in recent decades. For example, *Tas1rs*, consisting of three members, *Tas1r1*, *Tas1r2* and *Tas1r3*, are relatively highly conserved in almost all vertebrates [42]. A pseudogenized *Tas1r1* has been reported in the giant panda (*Ailuropoda melanoleuca*) and was suggested to coincide with the loss of the umami taste modality [43,44]. The chicken (*Gallus gallus*) has lost *Tas1r2* and thus may be insensitive to sweet compounds [42], whereas three vampire bats, the hairy-legged vampire bat (*Diphylla ecaudata*), common vampire bat (*Desmodus rotundus*), and white-winged vampire bat (*Diaemus youngi*), have lost both umami and sweet taste modalities [45,46]. Jiang et al. (2012) [31] reported that all three *Tas1rs* were lost in sea lion (*Zalophus californianus*) and the common bottlenose dolphin (*Tursiops truncatus*), which is consistent with their unique feeding behavior of swallowing food whole without chewing. *Tas2rs* are less conserved than *Tas1rs* [42]. The number of *Tas2rs* ranges from three in chicken to 69 in the guinea pig (*Cavia porcellus*), with an average number of ~30 in mammals [47,48], and the similarity of *Tas2rs* is approximately 30–70% [28]. By searching the dolphin genome (at 2.59 × coverage), Jiang et al. (2012) [31] demonstrated that dolphins have lost sweet, umami and bitter taste perception; however, they did not investigate the other two taste modalities, sour and salt. Li et al. (2014) [47] investigated *Tas2rs* gene repertoires in vertebrates, and they demonstrated that dietary toxins are a major selective force shaping the diversity of the *Tas2r* repertoire.

To investigate the cetacean taste system and to test hypotheses proposed in previous studies, we designed degenerate PCR primers to amplify all three *Tas1rs*, ten *Tas2rs*, *Pkd2l1*, and three ENaC members (ENaC α, β and γ) from cetacean genomes. Our results indicate that almost all cetaceans have lost sour, sweet, umami and most of the bitter taste modality, while the salt taste may be the only modality retained in cetaceans.

## Results and discussion

### Loss of sour, sweet, bitter and umami taste modalities in cetaceans

**Figure 1:**
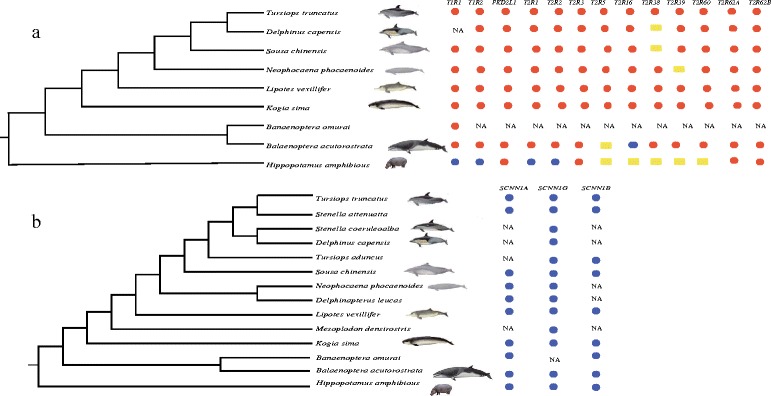
**The amplification of taste receptor genes in cetaceans and hippopotamus. a**: Degenerate PCR amplification of *Tas1r1*, *Tas1r2*, *Pkd2l1*, and 10 *Tas2rs* in cetaceans and hippopotamus. Red circles indicate pseudogenes, blue circles indicate functional genes, yellow boxes indicate unsuccessful amplification, and NA indicates no amplification was carried out. **b**: PCR amplification of three candidate salt taste receptor genes in cetaceans and hippopotamus. Blue circles indicate functional genes, and NA indicates no amplification was carried out.

We successfully amplified *Tas1r1*, *Tas1r2*, *Pkd2l1*, 10 bitter taste receptor genes (T*as2r1*, T*as2r2*, T*as2r3*, T*as2r5*, T*as2r16*, T*as2r38*, T*as2r39*, T*as2r60*, T*as2r62a* and T*as2r62b*), and three salt taste receptor genes (*scnn1a*, *scnn1b* and *scnn1g*) from major lineages of cetaceans (7–11 toothed whales and 1–2 baleen whales) and from Hippopotamidae (*Hippopotamus amphibious*) (Figures 1a, b, Additional file 1: Table S1 and Additional file 2: Tables S2-S7). These sequences were deposited in GenBank [GenBank: KJ524713-KJ524837]. Multiple ORF-disrupting indels and premature stop codons were identified in sour, sweet, bitter and umami taste receptor genes in all cetaceans. *Tas2r16* was intact in the baleen whale. We mapped these mutations and premature stop codons onto all the amplified gene sequences, except for *Tas2r62a* and *Tas2r62b*, because useful reference sequences were not available for these two genes (Additional file 3: Figures S1-S11). Furthermore, for Hippopotamidae, *Tas1r1*, *Tas1r2*, *Tas2r2* and *Tas2r3* were found to be intact, but *Pkd2l1*, *Tas2r1* and *Tas2r60* were pseudogenized. Based on the location of the first premature stop codon in the secondary structure of each protein, all these inactivation mutations are predicted to cause protein truncation (Additional file 4: Table S8). Although we have tried multiple primers to amplify *Tas1r3*, we failed to amplify even one exon eventually. Considering that both *Tas1r1* and *Tas1r2* had been identified as pseudogenes, we speculated that the umami and sweet tastes had been lost in the cetaceans. According to sequence alignments of the three salt taste receptor genes, we did not identify any inactivation mutations in salt taste receptor genes in cetaceans or Hippopotamidae. For *Tas1r1, Tas1r2*, *Pkd2l1*, and 10 *Tas2rs* genes, we only chose 7 cetacean species to represent most major cetacean lineages. Considering that we have identified some shared indels and/or premature stop codons in these genes of some cetacean lineages, it is reasonable to make a conclusion that these genes might have become pseudogenized in cetaceans. However, it is necessary to pay more attention to sensory perception of cetaceans in the future, particularly using high-throughput DNA sequencing techniques and sampling more genes in more species.

Six exons of *Tas1r1* and *Tas1r2* stretching from nearly the beginning of the N-terminus to the end of the C-terminus were amplified in the major cetacean lineages and Hippopotamidae (Additional file 2: Tables S2-S3). PKD2L1 is composed of an intracellular N-terminal region, a six transmembrane domain and an intracellular C-terminal region [49]. A total of 15 *Pkd2l1* exons were amplified in representative cetacean branches and Hippopotamidae (Additional file 2: Table S4). Bitter compounds are perceived by numerous intronless *Tas2rs* [28-30]. Jiang et al. (2012) [31] have identified 10 *Tas2rs* by searching dolphin’s genome, and showed dolphin may have lost bitter taste perception owing to pseudogenization of these 10 *Tas2rs*. Here we successfully amplified these 10 *Tas2r* genes, ranging from 872 to 1,161 bp and used these to explore the evolution of *Tas2rs* in extant cetaceans and Hippopotamidae (Additional file 1: Table S1).

Based on sequence alignments against cow (*Bos taurus*) homologues, we identified multiple ORF-disrupting indels and premature stop codons in *Tas1r1*, *Tas1r2*, *Pkd2l1* and in ten *Tas2rs* scattered among cetacean branches. We also found ORF-disrupting mutations in *Pkd2l1* and in two bitter taste receptor genes (*Tas2r*3 and *Tas2r60*) in Hippopotamidae (Additional file 3: Figures S1-S11). All these inactivating mutations were mapped onto the species tree (Additional file 5: Figures S12-S22), and the locations of the first premature stop codons are listed in Additional file 4: Table S8. For *Tas1r1*, we identified a premature stop codon shared by all cetaceans, a 5 bp deletion shared by all toothed whales and a 17 bp deletion in two baleen whales (Additional file 3: Figure S2). For *Tas1r2,* a 5 bp deletion was found on the stem Odontoceti (Additional file 3: Figure S3), suggesting that the pseudogenization event had happened in the common ancestor of the Odontoceti. The ability to sense sour-taste substances is important for protecting mammals from ingesting toxic food. For PKD2L1, the sole candidate sour taste receptor, we found two premature stop codons shared by all toothed whales, excluding the Dwarf sperm whale (*Kogia sima*) and a premature TGA stop codon shared by all cetaceans except for the baiji (Additional file 3: Figure S1). Interestingly, the ninth exon of *Pkd2l1* was lost in the finless porpoise (*Neophocaena phocaenoides*) (Additional file 3: Figure S1), which was confirmed by an additional eight individuals.

We amplified 10 *Tas2rs*, including *Tas2r1*-*3*, *5*, *16*, *38*–*39*, *60*, *62a* and *Tas2r62b* in cetaceans and in five members of them in Hippopotamidae members (Additional file 1: Table S1). Compared with corresponding functional sequences of *Tas2r1*, we found a 1 bp deletion in three species of Delphinidae, a premature stop codon (TGA) in all cetaceans except for the Dwarf sperm whale, and another premature stop codon (TGA) in four toothed whales (Additional file 3: Figure S4). In *Tas2r2*, *Tas2r5* and *Tas2r16*, we found shared ORF-disrupting mutations and/or premature stop codons in all toothed whales, such as a 1 bp or a 2 bp deletion in *Tas2r2* (Additional file 3: Figure S5), a shared TGA premature stop codon in *Tas2r5* (Additional file 3: Figure S7), and a 4 bp deletion in *Tas2r16* (Additional file 3: Figure S8), suggesting that the functional loss of these genes happened in the common ancestor of toothed whales. However, we could not exclude the possibility that the pseudogenization event in *Tas2r2* might have occurred in the ancestor of all cetaceans, although we failed to amplify the whole *Tas2r2* sequence in the common minke whale (*Balaenoptera acutorostrata*). Most interestingly, the common minke whale still had an intact *Tas2r16*, but whether it is still functional requires further investigation*.* Shared inactivating mutations in *Tas2r3*, *Tas2r38*, *Tas2r39* and *Tas2r60* were all successfully mapped on the stem cetaceans, although *Tas2r38* was successfully amplified in only five species. A 1 bp deletion in *Tas2r3* (Additional file 3: Figure S6), a 2 bp deletion in *Tas2r38* (Additional file 3: Figure S9), a 4 bp insertion in *Tas2r39* (Additional file 3: Figure S10), and a shared TGA premature stop codon in *Tas2r60* (Additional file 3: Figure S11) were identified, suggesting that the functional loss happened in the common ancestor of the cetaceans. For *Tas2r62a* and *Tas2r62b*, we could not definitively identify indels because the homologous gene in cow is a pseudogene and in dog only a portion of the gene has been reported and may, therefore, not be functional. However, we are confident that both *Tas2r62a* and *Tas2r62b* in cetaceans are pseudogenes because correct translation reveals multiple premature stop codons. For the three pseudogenized *Tas2rs* of Hippopotamidae, no shared ORF-disrupting mutation was found between cetaceans and Hippopotamidae, suggesting independent pseudogenization events in cetaceans and Hippopotamidae.

### Relaxation of selective pressure on taste genes

To evaluate the selective pressure on these pseudogenized taste receptor genes in cetaceans, the ratios of nonsynonymous to synonymous substitutions (*d*_N_/*d*_S_) were calculated (Table 1). For *Tas1r1*-*2* and *Pkd2l1*, based on the assumption that all branches had a single ω value, purifying selection was seen across the tree for the three genes according to comparison between model A and model B (ω = 0.2919, *p* = 0; ω = 0.20585, *p* = 0; ω = 0.28788, *p* = 0; respectively). Further comparison between model A and model C in which pseudogenized branches had a ω_2_ while other branches had a ω_1_ showed that ω in pseudogenized branches was significantly higher for umami, sweet, and sour taste receptor genes (ω_1_ = 0.25599, ω_2_ = 0.68390 in model C, *p* = 2.82E-12 in dataset I; ω_1_ = 0.17096, ω_2_ = 0.49085 in model C, *p* = 7.97E-14 in dataset II; ω_1_ = 0.24058, ω_2_ = 0.55166 in model C, *p* = 1.35E-07 in dataset III), indicating that functional constraint was slightly relaxed in cetaceans for *Tas1r1*and *Tas1r2* and in cetaceans plus Hippopotamidae for *Pkd2l1*. To further evaluate whether selective pressure was completely removed, we performed comparisons between model C and model D which had a fixed ω_2_ = 1 in pseudogenized branches. This analysis showed that functional constraints on *Tas1r1*and *Tas1r2* were not completely removed in cetaceans nor on *Pkd2l1* in cetaceans plus Hippopotamidae (*p* = 0.01 in model C vs D of dataset I; *p* = 9.90E-07 in model C vs D of dataset II; *p* = 5.45E-05 in model C vs D of dataset III). Finally, model E, which allowed different branches their own ω was significantly fixed the data than model C (*p* = 2.33E-05 in model C vs E of dataset I; *p* = 2.41E-05 in model C vs E of dataset II; *p* = 6.78E-07 in model C vs E of dataset III), indicative of variable ω across the tree for the three genes.

**Table 1:** **Likelihood ratio tests of various models on the selective pressures on** ***Tas1r1*** **,** ***Tas1r2*** **,** ***Pkd2l1*** **, and** ***Scnn1g***

	**Models**	**ω**	**-lnL**	**np**	**Models compared**	**2Δ (ln L)**	***p*** **-value**
Dataset I:	*Tas1r1*						
	All branches have one ω (A)	0.2919	12711.16	28			
	All branches have one ω = 1(B)	1	13033.12	27	B vs. A	643.92	0
	The branches with pseudogenized *Tas1r1* has ω_2_, others have ω_1_ (C)	ω_1_ = 0.25599	12686.76	29	A vs. C	48.81	2.82E-12
		ω_2_ = 0.68390					
	The branches with pseudogenized *Tas1r1* has ω_2_ = 1, others have ω_1_ (D)	ω_1_ = 0.25589	12690.33	28	D vs. C	7.15	0.01
		ω_2_ = 1.00000					
	Each branch has its own ω(E)	Variable ω by branch	12655.22	53	C vs. E	63.06	2.33E-05
Dataset II:	*Tas1r2*						
	All branches have one ω (A)	0.20585	11866.17	24			
	All branches have one ω = 1(B)	1	12287.21	23	B vs. A	842.08	0
	The branches with pseudogenized *Tas1r2* has ω_2_, others have ω_1_ (C)	ω_1_ = 0.17096 ω_2_ = 0.49085	11838.26	25	A vs. C	55.81	7.97E-14
	The branches with pseudogenized *Tas1r2* has ω_2_ = 1, others have ω_1_ (D)	ω_1_ = 0.16972 ω_2_ = 1.00000	11850.23	24	D vs. C	23.95	9.90E-07
	Each branch has its own ω(E)	Variable ω by branch	11809.99	45	C vs. E	56.54	2.41E-05
Dataset III:	*Pkd2l1*						
	All branches have one ω(A)	0.28788	8723.86	28			
	All branches have one ω = 1(B)	1	8907.34	27	B vs. A	366.97	0
	The branches with pseudogenized *Pkd2l1* has ω_2_, others have ω_1_ (C)	ω_1_ = 0.24058 ω_2_ = 0.55166	8709.97	29	A vs. C	27.79	1.35E-07
	The branches with pseudogenized *Pkd2l1* has ω_2_ = 1, others have ω_1_ (D)	ω_1_ = 0.23991	8718.11	28	D vs. C	16.29	5.45E-05
		ω_2_ = 1.00000					
	Each branch has its own ω (E)	Variable ω by branch	8673.30	53	C vs. E	73.33	6.78E-07
Dataset IV:	*Scnn1g*						
	All branches in cetaceans have a ω_3_, other branches have a ω_2_. (F)	ω_2_ = 0.19790	8999.60	46			
		ω_3_ = 1.05041					
	M2a-rel: All branches in cetaceans have a ω_3_, other branches have a ω_2_, ω_2_ = ω_3_. (G)	ω_2_ = ω_3_ = 0.25665	9036.40	45	F vs G	73.58	0
	Site model						
	M1a (nearly neutral)	ω_0_ = 0.05745 ω_1_ = 1.00000	9068.93	43			
	M2a (positive selection)	ω_0_ = 0.05745 ω_1_ = 1.00000 ω_2_ = 1.00000	9068.93	45	M1a VS M2a	0	1
	M8a	ω = 1.00000	9037.05	44			
	M8	ω = 1.35413	9036.59	45	M8a VS M8	0.93	0.33

We analyzed seven bitter taste receptor genes, excluding *Tas2r38*, *Tas2r62a* and *Tas2r62b* because the species from which we successfully amplified *Tas2r38* were scarce, and we only retrieved pseudogenes as query sequences for *Tas2r62a* and *Tas2r62b*. The analysis process was similar to that for *Tas1r1*, *Tas1r2* and *Pkd2l1*, and found that the functional constraints were almost completely removed from these seven *Tas2rs* (Additional file 6: Tables S9-S15).

The shift of habitat from land to water approximately 52.5 Mya and subsequent changes in feeding behavior and habitat might have contributed to the loss of the four tastes in cetaceans. For example, basal cetaceans have several suites of synapomorphies, including reduction of the crushing basins of teeth, which suggested a major change of dental function, and development of the long and narrow postorbital and temporal region of the skull in early cetaceans. Those synapomorphies could affect sense organs and may be related to dietary changes in early cetaceans [50,51]. Alongside living in aquatic water environments, cetaceans have evolved unique feeding behaviors including the swallowing of food without chewing in toothed whales and filtering in baleen whales [52,53]. These behaviors further reduced their dependence on taste in the search for food resources. Anatomical evidence also reveals that both toothed and baleen whales have degenerated tongue epithelia containing only few taste buds [54-56]. The tongues of the Pacific whitesided dolphin (*Lagenorhynchus obliquidens*), bottlenose dolphin, striped dolphin (*Stenella coeruleoalba*), and baiji have been reported to lack circumvallate papilla, foliate papilla and fungiform papilla [54-56]. Furthermore, most cetaceans live in oceans where high concentrations of sodium might mask other taste modalities. This would further decrease their dependence on taste for seeking out prey, leading to the loss of basic taste modalities.

It is noteworthy that there are many reports detailing the distribution of Tas1rs and Tas2rs in non-oral cavities, including intestinal tract [57-60], respiratory tract [61-63], pancreas [64] and brain [65,66], and in these non-oral cavities these taste receptors can also interact with taste substances but they induce different reactions. There are also other receptors that detect small peptides and amino acids such as metabotropic glutamate receptors (mGluRs) and calcium-sensing receptors (CaSRs) [67-70]. We, therefore, cannot exclude the possibility that cetaceans may retain some umami taste despite Tas1r1 being pseudogenized. It will be interesting to investigate other candidate umami taste receptors to see whether cetaceans have completely lost the umami taste.

### Salt taste is the sole functional taste modality retained in cetaceans

The sense of salt taste can contribute to the ingestion of Na^+^ and other minerals. It is widely believed that the epithelial sodium channel (ENaC), composed of three homologous ENaCα, β and γ subunits, plays a crucial role in the perception of salt taste [15,71,72]. Belonging to the ENaC/degenerin family, ENaC was proposed to be an α-γ-β trimer based on the structure of ASIC1 (acid sensing ion channel 1), which is another member of the ENaC/degenerin family [73]. Each ENaC subunit consists of an intracellular N terminal region, an intracellular C terminal region, and a two-transmembrane domain [72]. Here, we successfully amplified ENaCα, β and γ subunits encoded by *scnn1a*, *scnn1b* and *scnn1g*, respectively, in representative toothed and baleen whales and in Hippopotamidae (Additional file 1: Table S1 and Additional file 2: Tables S5-S7).

**Figure 2:**
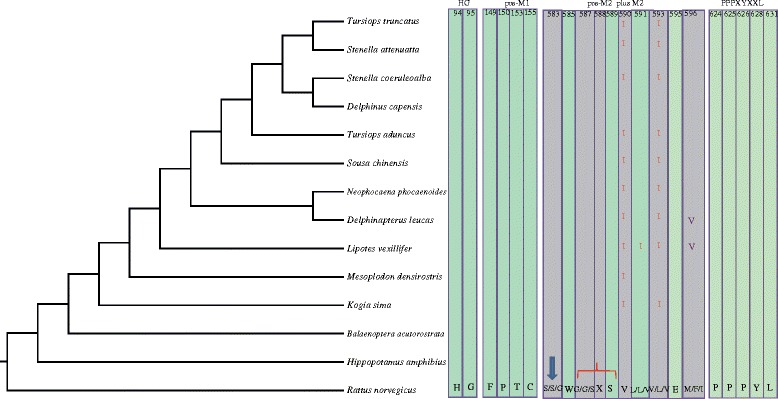
**Variations in three salt taste receptor genes.** Variations in all completely and partially (80%) conserved residues are shown with respect to rat (*Rattus norvegicus*) homologous sequence. Light green bar indicates completely conserved sites, and light purple bar indicates 80% or greater conserved sites. Black words below indicate conserved residues in rat ENaCα, β and γ, respectively, if the residues are the same in the three genes, we use only one symbol. Numbers indicate the location of residues in rat ENaCα. Residues in red indicate variations in ENaCγ, while residues in dark purple indicate variations in ENaCα. Bracket denotes selective sites, and arrow denotes amiloride binding site.

No inactivating mutation was identified in any of these three genes. Furthermore, we have identified multiple conserved residues in cetaceans that are essential for channel function. These conserved residues reside in motifs that include the conserved proline-rich motifs containing PPPXYXXL residues in the C-terminus, HG residues in the N-terminus, FPXXTXC in post-M1 (first transmembrane domain), completely conserved residues in the second transmembrane domain (M2), and conserved Cys-rich domains in the extracellular loop [74]. All of these conserved motifs are essential for channel function, for example post-M2 and M2 constitute the outer pore entry and selectivity filter [74], conserved HG in the N-terminus plays an important role in gating [75], Cys-rich domains are vital for tertiary structure of the extracellular loop [76], and conserved proline-rich motifs in the C terminus take part in channel ubiquitination, endocytosis, and degradation of the ENaC [77,78]. Based on sequence alignments, *Scnn1a*, *Scnn1b* and *Scnn1g* possessed all these conserved amino acids, except for a conserved HG in the N-terminus of *Scnn1a*, a conserved FPXXTXC in post-M1, and two important residues in the Cys-rich domains of *Scnn1b*; however these omissions are likely to be because of our incomplete gene amplification. Interestingly, we identified a variable residue, γV591I, in the completely conserved M2 motif in the baiji. Among residues with 80% or greater conservation in M2, we identified a γV590I variation in toothed whales, a γV593I variation in all toothed whales except for the beaked whale (*Mesoplodon densirostris*), and a αM596V variation in cetaceans (Figure 2). These substitutions probably affect the formation of the channel pore based on their distribution in pre-M2 and M2, which are known to participate in the formation of the channel pore. Even though we could not identify all conserved sequences owing to incomplete amplification, the above analyses strongly suggested that the salt taste genes were intact.

We used site models (m1a vs m2a; m8 vs m8a), and branch site model to test whether salt taste receptor genes were under positive selection, and Clade model C (compared with m2a_ rel, a null model for Clade model C) was used to identify divergent selection acting on different clades [79,80]. For *scnn1g*, Clade model C marked all cetaceans as foreground branches, with ω_3_ = 1.05041 for the foreground branch and ω_2_ = 0.19790 for the background branch (F vs G of dataset IV in Table 1), which is significantly better than m2a_rel (*p* = 0). Our clade model showed evidence of significant divergent selection, and the ω in cetacean was greater than one, suggesting positive selection of this gene in cetacean clade. M8 in the site model also identified some positively selected sites, although the model was not significantly better than the null model (*p* = 0.33) and the *p*-values of the sites were less than 95% (Dataset IV in Table 1). Using the latest model FUBAR [81], we also identified four pervasive diversifying selection sites at posterior probability ≥0.8 (data not shown). Because amino acid substitution affects proteins by altering their physicochemical properties and structure, we employed a complementary protein-level approach implemented in TreeSAAP [82]. Our TreeSAAP analysis identified four significant physicochemical changes owing to amino acid residues changes in ENaCγ: equilibrium constant (ionization of COOH), isoelectric point, power to be at the C-terminal and tendency to form alpha-helix (Additional file 7: Table S16). Selective pressure analysis of ENaCα and ENaCβ failed to identify positive signatures, suggesting that *scnn1a* and *scnn1b* are still under strong purifying selection (data not shown). Our TreeSAAP analysis identified eleven and five significant physicochemical amino acid changes in ENaCα and β, respectively (Additional file 7: Table S16). These significant changes may contribute to cetaceans’ adaptation by increasing ENaC activities.

ENaC is widely distributed in tissues associated with Na^+^ transport, including kidney, distal colon, lung, sweat ducts, salivary ducts and skin, and plays vital roles in these tissues [41,72]. In lung, the main function of ENaC is not only in ion and water homeostasis, but also in maintaining the appropriate level of hydration of the fluid layer [83]. ENaCα knockout mice die within a few days after birth because they fail to clear fetal lung liquid [84]. In the kidney and distal colon, ENaC is vital for the homeostasis of blood K^+^ and Na^+^ levels, especially in the kidney where channels have an important role in overall Na^+^ balance [83]. Mutations in the conserved HG motifs cause a renal salt-wasting syndrome called pseudohypoaldosteronism type 1 (PHA-1) [85], and mutations in the conserved PPPXY motif in β- and γ-ENaC subunits are associated with Liddle’s syndrome, a form of monogenic hypertension [86,87]. Cetaceans living in the hyperisotonic marine environment have to overcome the problems caused by high concentrations of sodium in the water. The osmotic pressure of urine is higher than the vascular osmotic pressure in cetaceans [88-92]. Thus, the importance of ENaC in ion and water homeostasis and in maintaining the appropriate level of hydration of the fluid layer may have provided selective pressure to preserve salt taste receptor function in cetaceans. This is probably related to their distribution in the kidney especially in cortical collecting tubes and to their function in Na^+^ reabsorption [83]. Taking into consideration the degenerated tongue epithelia in cetaceans and the importance of ENaC in the kidney and other organs, we propose that the intact of ENaC may be owed to its function in kidneys and other organs. Whether cetaceans can taste salt is still unknown; the answer to this question awaits further investigation.

## Conclusions

Receptor genes for the five specific tastes were investigated among the major cetaceans and the five basic taste modalities were assessed in marine mammals. Cetaceans appear to have lost four basic taste modalities including sour, sweet, umami, and the majority of the bitter taste sensation. However, as for umami taste, there are also other receptors that detect small peptides and amino acids, making it necessary to detect other candidate genes of umami to further reveal the evolution pattern of cetacean umami receptors. The integrity of salt receptor genes in all cetaceans studied here, may be owed to their function in Na^+^ reabsorption, which is key to osmoregulation during aquatic adaptation.

## Methods

### Polymerase chain reaction and DNA sequencing

Genomic DNA was extracted from muscle and/or blood samples from representative cetaceans including toothed and baleen whales, and the hippo (*Hippopotamus amphibious*) using a standard phenol1chloroform protocol [93]. Cetacean samples were collected from stranded or incidentally captured/killed animals in coastal China Seas by our lab members; therefore, ethical approval has not been requested. When the aquatic mammals were reported to be stranded or incidental captured/killed, we contacted local Oceanic and Fisheries Bureaus which perform conservation and management of aquatic animals on behalf of Chinese government. Once we got their permissions, we went to the sites of stranding or incidental catching to collect animal tissue samples for research purpose. The hippo sample was a piece of muscle sampled from an died individual, which was provided to us by Chengdu Zoo, Sichuan Province, China for the present study, and we have permission from this institution to conduct our experiments on this sample. Voucher specimens were preserved at Nanjing Normal University. Based on alignments of homologous taste receptor genes for the five taste modalities between the common bottlenose dolphin (http://asia.ensembl.org/Tursiops_truncatus/Info/Index) and cattle (http://asia.ensembl.org/Bos_taurus/Info/Index), a series of degenerate primers were designed (Additional file 8: Table S17). PCR reactions (30 μl) contained 0.8 μl genomic DNA, 1 unit of Taq polymerase (Takara), 0.2 μmol of each primer, 3 μl of 10 × PCR buffer, 0.2 μmol of dNTP and 2.5 μmol of MgCl_2_. Cycling parameters were as follows: denaturation at 95°C for 5 min, then 35 cycles of 95°C for 30 s, 55–58°C for 40 s, 72°C for 40 s, and finally an elongation at 72°C for 10 min. The amplified PCR products were separated by agarose gel electrophoresis and gel-purified products were cloned into pMD18-T (Takara). PCR products were sequenced in both directions using an ABI PRISM 3730 DNA Sequencer. Three to five clones for each gene or pseudogene were obtained to confirm its sequence. Other sequences used in analyses were downloaded from the Ensembl Genome database (http://www.ensembl.org) and GenBank (http://www.ncbi.nlm.nih.gov) with accession numbers listed in Additional file 9: Table S18. The secondary structures of proteins were estimated using TMHMM (http://www.cbs.dtu.dk/services/TMHMM/).

### Phylogenetic reconstruction

To access sequence variability among different species, we used CLUSTAL W [94] in MEGA5 [95] to conduct sequence alignments. To analyze selective pressure, CODEML in PAML v4.4 [79] was used, and we incorporated the widely accepted phylogenetic trees of cetaceans [2,96-98]. For genes with intact open reading frames, nucleotide sequence alignments were conducted based on protein sequence alignment, while for pseudogenes we selected closely related functional sequences as queries to ascertain indels and premature stop codons. In addition, we used the TreeSAAP 3.2 software package [82] to detect significant physicochemical amino acid changes among residues in three ENaC members. The software program TreeSAAP measures the selective influences on 31 structural and biochemical amino acid properties during cladogenesis, and performs goodness-of-fit and categorical statistical tests [82]. Within TreeSAAP, magnitudes of non-synonymous changes are classified into eight categories according to the change in specific physicochemical properties, in which 1–3 are conservative while 6–8 are radical. After running, a z-score was generated for each category, with a positive z-score meaning that a given region is under positive selection influence. Here, we only considered amino acid properties with significant positive z-scores in categories 6–8 to be under positive selection, and a sliding window of 15 was performed.

## Availability of supporting data section

The data sets supporting the results of this article are available in the Dryad repository, http://dx.doi.org/10.5061/dryad.7qp63 [99]. This repository contains all of the datasets from Table 1.

## Additional files

Additional file 1: Table S1. Degenerate PCR amplification of five taste-related genes among representative cetaceans and hippopotamus. Note: tick represents successfully amplified. Additional file 2: Tables S2-S7. Statistics for amplified exons from each taste receptor gene for each species. Note: tick represents successfully amplified. Additional file 3: Figures S1-S11. Indels and premature stop codons in *Pkdl21*, *Tas1r1, Tas1r2, Tas2r1-3, Tas2r5, Tas2r16, Tas2r38-39, and Tas2r60.* Indels are highlighted in red, while premature stop codons are indicated in green. Additional file 4: Table S8. The location of the first premature stop codon in each pseudogenized taste receptor gene. N represents the N-terminus, FEL represents the First extracellular loop, SEL represents Second extracellular loop, TEL represents the Third extracellular loop, FIL represents the Fourth intracellular loop, TM represents transmembrane domain, SIL represents Second intracellular loop, and NA represents non-amplification in our analysis. Additional file 5: Figures S12-S22. Indels and premature stop codons mapped on the species tree for pseudogenized taste receptor genes. A list of phylogenetic trees of the taste receptor genes analyzed in the present study with all indels and premature stop codons mapped in order. All indels are characterized with bars, with each color representing a different indel and the same color representing the same indel across the tree. Premature stop codons are characterized with ellipses; we did not differentiate premature stop codons and all have the same color. Additional file 6: Tables S9-S15. Likelihood ratio tests of various models on the selective pressures on seven bitter taste receptor genes. Additional file 7: Table S16. Physicochemical properties under positive destabilizing selection in ENaCα, β, γ. Additional file 8: Table S17. PCR primers for each taste receptor gene. Additional file 9: Table S18. Accession numbers for species used in PAML analysis.
