# Supplementary material for: The loss of taste genes in cetaceans
Source: BMC Evol Biol. 2014 Oct 12;14:218. doi: 10.1186/s12862-014-0218-8 (PMC4232718; doi:10.1186/s12862-014-0218-8)
Supplement: Additional file 1: Table S1. — Degenerate PCR amplification of five taste-related genes among representative cetaceans and hippopotamus. Note: tick represents successfully amplified. [file 12862_2014_218_MOESM1_ESM.doc]

|  | *Tas1r1* | *Tas1r2* | *pkd2l1* | *Tas2r1* | *Tas2r2* | *Tas2r3* | *Tas2r5* | *Tas2r16* | *Tas2r38* | *Tas2r39* | *Tas2r60* | *Tas2r62a* | *Tas2r62b* | *scnn1a* | *scnn1b* | *scnn1g* |
| --- | --- | --- | --- | --- | --- | --- | --- | --- | --- | --- | --- | --- | --- | --- | --- | --- |
| *Tursiops truncatus* | √ | √ | √ | √ | √ | √ | √ | √ |  | √ | √ | √ | √ | √ | √ | √ |
| *Stenella attenuatta* |  |  |  |  |  |  |  |  |  |  |  |  |  | √ | √ | √ |
| *Sousa chinensis* | √ |  | √ | √ | √ | √ | √ | √ |  | √ | √ | √ | √ | √ | √ | √ |
| *Neophocaena phocaenoides* | √ | √ | √ | √ | √ | √ | √ | √ | √ |  | √ | √ | √ | √ | √ | √ |
| *Lipotes vexillifer* | √ | √ | √ | √ | √ | √ | √ | √ | √ | √ | √ | √ | √ | √ | √ | √ |
| *Banaenoptera omurai* | √ |  |  |  |  |  |  |  |  |  |  |  |  | √ | √ |  |
| *Balaenoptera acutorostrata* | √ | √ | √ | √ | √ | √ |  | √ | √ | √ | √ | √ | √ | √ | √ | √ |
| *Kogia sima* | √ | √ | √ | √ | √ | √ | √ | √ | √ | √ | √ | √ | √ | √ | √ | √ |
| *Hippopotamus amphibious* | √ | √ | √ | √ | √ | √ |  |  |  |  | √ | √ |  | √ | √ | √ |
| *Delphinus capensis* |  | √ | √ | √ | √ | √ | √ | √ | √ |  | √ | √ | √ | √ |  | √ |
| *Mesoplodon densirostris* |  |  |  |  |  |  |  |  |  |  |  |  |  |  |  | √ |
| *Delphinapterus leucas* |  |  |  |  |  |  |  |  |  |  |  |  |  |  |  | √ |
| *Stenella coeruleoalba* |  |  |  |  |  |  |  |  |  |  |  |  |  |  |  | √ |

Table S1. **Table S1 Degenerate PCR amplification of five taste-related genes among representative cetaceans and hippopotamus.** Note: tick represents successfully amplified.
