# Supplementary material for: The loss of taste genes in cetaceans
Source: BMC Evol Biol. 2014 Oct 12;14:218. doi: 10.1186/s12862-014-0218-8 (PMC4232718; doi:10.1186/s12862-014-0218-8)
Supplement: Additional file 2: Tables S2-S7. — Statistics for amplified exons from each taste receptor gene for each species. Note: tick represents successfully amplified. [file 12862_2014_218_MOESM2_ESM.doc]

**Tables S2-S7 Statistics for amplified exons from each taste receptor gene for each species.**

Note: tick represents successfully amplified.

Tas1r1

|  | Exon 1 | Exon 2 | Exon 3 | Exon5 | Exon 6 | Exon7 |
| --- | --- | --- | --- | --- | --- | --- |
| *Tursiops truncatus* | √ | √ | √ | √ | √ | √ |
| *Sousa chinensis* | √ | √ | √ | √ | √ | √ |
| *Neophocaena phocaenoides* | √ | √ | √ | √ | √ | √ |
| *Lipotes vexillifer* | √ | √ | √ | √ | √ | √ |
| *Kogia sima* | √ | √ | √ | √ | √ | √ |
| *Balaenoptera acutorostrata* | √ | √ | √ | √ | √ | √ |
| *Banaenoptera omurai* | √ | √ | √ | √ | √ | √ |
| *Hippopotamus amphibious* | √ | √ | √ | √ | √ | √ |

Tas1r2

|  | Exon 1 | Exon 2 | Exon 3 | Exon 4 | Exon 5 | Exon 6 |
| --- | --- | --- | --- | --- | --- | --- |
| *Tursiops truncatus* | √ | √ | √ | √ | √ | √ |
| *Delphinus capensis* | √ | √ | √ | √ | √ | √ |
| *Neophocaena phocaenoides* | √ | √ | √ | √ | √ | √ |
| *Lipotes vexillifer* | √ | √ | √ | √ | √ | √ |
| *Kogia sima* | √ | √ | √ | √ | √ | √ |
| *Balaenoptera acutorostrata* | √ | √ | √ | √ | √ | √ |
| *Hippopotamus amphibious* |  | √ | √ | √ | √ | √ |

Pkd2l1

|  | Exon 1 | Exon 2 | Exon3 | Exon  4 | Exon5 | Exon6 | Exon7 | Exon  8 | Exon9 | Exon 10 | Exon11 | Exon  12 | Exon 13 | Exon 15 | Exon 16 |
| --- | --- | --- | --- | --- | --- | --- | --- | --- | --- | --- | --- | --- | --- | --- | --- |
| *Tursiops truncatus* | √ | √ | √ | √ | √ | √ | √ | √ | √ | √ | √ | √ | √ | √ | √ |
| *Delphinus capensis* | √ | √ | √ | √ | √ | √ | √ | √ | √ | √ | √ | √ | √ | √ | √ |
| *Sousa chinensis* | √ | √ | √ | √ | √ | √ | √ | √ | √ | √ | √ | √ | √ | √ | √ |
| *Neophocaena phocaenoides* | √ | √ | √ | √ | √ | √ | √ | √ | √ | √ | √ | √ | √ | √ | √ |
| *Lipotes vexillifer* | √ | √ | √ | √ | √ | √ | √ | √ | √ | √ | √ | √ | √ | √ | √ |
| *Balaenoptera acutorostrata* | √ | √ | √ | √ | √ | √ | √ | √ | √ | √ | √ | √ | √ |  |  |
| *Kogia sima* | √ |  | √ | √ | √ | √ | √ | √ | √ | √ | √ | √ | √ | √ | √ |
| *Hippopotamus amphibious* |  | √ | √ | √ | √ | √ | √ | √ | √ | √ | √ | √ | √ | √ | √ |

Scnn1a

|  | Exon 1 | Exon 2 | Exon3 | Exon  4 | Exon  5 | Exon  6 | Exon  7 | Exon  8 | Exon  9 | Exon  10 | Exon  11 | Exon  12 -14 |
| --- | --- | --- | --- | --- | --- | --- | --- | --- | --- | --- | --- | --- |
| *Tursiops truncatus* | √ | √ | √ | √ | √ | √ |  | √ | √ | √ | √ | √ |
| *Tursiops aduncus* | √ | √ | √ | √ | √ | √ | √ | √ | √ | √ | √ | √ |
| *Lipotes vexillifer* | √ | √ | √ | √ | √ | √ | √ | √ | √ | √ | √ | √ |
| *Balaenoptera acutorostrata* | √ |  | √ | √ |  | √ | √ | √ | √ | √ | √ |  |
| *Banaenoptera omurai* |  |  | √ |  |  | √ | √ | √ | √ | √ | √ |  |
| *Sousa chinensis* | √ |  | √ | √ | √ | √ | √ | √ | √ | √ | √ | √ |
| *Stenella attenuatta* | √ | √ | √ | √ |  | √ | √ | √ | √ | √ | √ | √ |
| *Delphinapterus leucas* | √ |  | √ | √ | √ | √ | √ | √ | √ | √ | √ | √ |
| *Neophocaena phocaenoides* | √ | √ | √ | √ |  | √ | √ | √ | √ | √ | √ | √ |
| *Kogia sima* | √ | √ | √ | √ | √ | √ | √ | √ | √ | √ | √ | √ |
| *Hippopotamus amphibious* | √ |  | √ |  |  | √ | √ | √ | √ | √ | √ |  |

Scnn1g

|  | Exon 1 | Exon 2 | Exon3 | Exon4 | Exon5 | Exon6 | Exon7 | Exon  8 | Exon9 | Exon 10 | Exon11 | Exon12 |
| --- | --- | --- | --- | --- | --- | --- | --- | --- | --- | --- | --- | --- |
| *Tursiops truncatus* | √ | √ | √ | √ | √ | √ |  | √ | √ | √ | √ | √ |
| *Delphinus capensis* | √ | √ | √ | √ | √ | √ | √ | √ | √ | √ | √ |  |
| *Sousa chinensis* | √ | √ | √ | √ | √ | √ | √ | √ | √ | √ | √ | √ |
| *Neophocaena phocaenoides* | √ | √ | √ | √ | √ | √ | √ | √ | √ | √ | √ | √ |
| *Lipotes vexillifer* | √ | √ | √ | √ | √ | √ | √ | √ | √ | √ | √ | √ |
| *Balaenoptera acutorostrata* | √ | √ | √ | √ | √ | √ | √ | √ | √ | √ | √ | √ |
| *Kogia sima* |  | √ | √ | √ | √ | √ | √ | √ | √ | √ | √ | √ |
| *Hippopotamus amphibious* |  | √ | √ | √ |  | √ | √ | √ | √ | √ |  | √ |
| *Mesoplodon densirostris* | √ | √ | √ | √ |  | √ | √ | √ | √ | √ | √ | √ |
| *Delphinapterus leucas* | √ | √ | √ | √ | √ | √ | √ | √ | √ |  | √ | √ |
| *Stenella coeruleoalba* | √ | √ | √ | √ | √ | √ | √ | √ | √ | √ | √ | √ |
| *Stenella attenuatta* | √ | √ | √ | √ | √ | √ | √ | √ | √ | √ | √ | √ |

***Scnn1b***

|  | Exon 3 | Exon 4 | Exon5 | Exon 6 | Exon 7 | Exon 8 | Exon 9 | Exon 12 |
| --- | --- | --- | --- | --- | --- | --- | --- | --- |
| *Lipotes vexillifer* | √ | √ | √ | √ | √ | √ | √ | √ |
| *Balaenoptera acutorostrata* | √ | √ | √ | √ |  | √ | √ | √ |
| *Banaenoptera omurai* | √ | √ | √ | √ | √ | √ |  |  |
| *Sousa chinensis* | √ | √ | √ | √ | √ | √ | √ |  |
| *Stenella attenuatta* | √ | √ | √ | √ |  | √ | √ | √ |
| *Tursiops truncatus* | √ | √ | √ | √ | √ | √ | √ | √ |
| *Neophocaena phocaenoides* | √ | √ | √ | √ | √ | √ | √ | √ |
| *Kogia sima* | √ | √ | √ | √ | √ | √ |  |  |
| *Hippopotamus amphibious* | √ |  |  |  |  | √ |  |  |
