# Supplementary material for: The loss of taste genes in cetaceans
Source: BMC Evol Biol. 2014 Oct 12;14:218. doi: 10.1186/s12862-014-0218-8 (PMC4232718; doi:10.1186/s12862-014-0218-8)

**Fig. S1: Indels and premature stop codons in *pkdl21*.** Indels were highlighted in red, while premature stop codon were indicated in green.
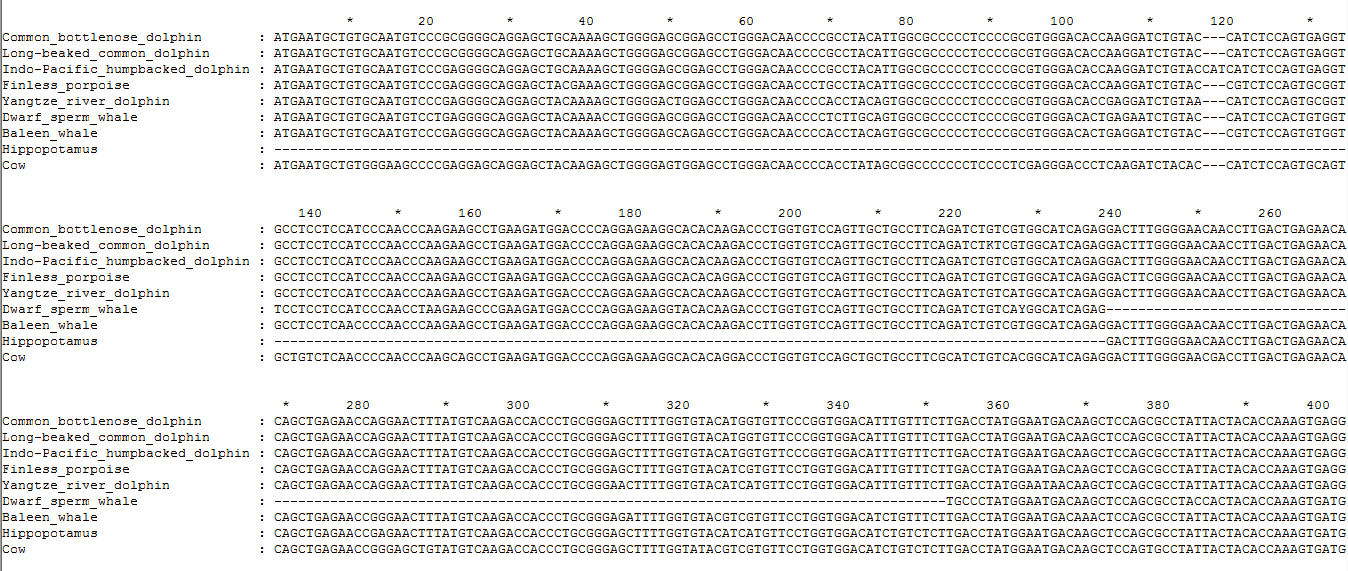

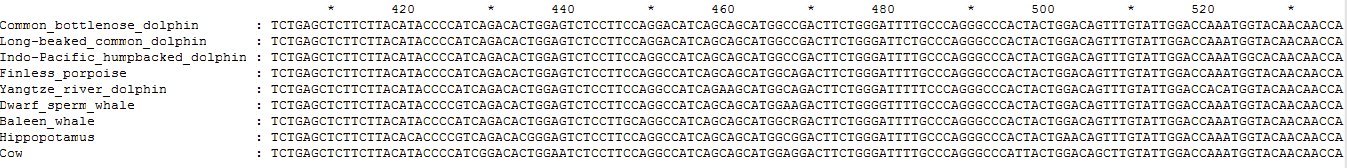


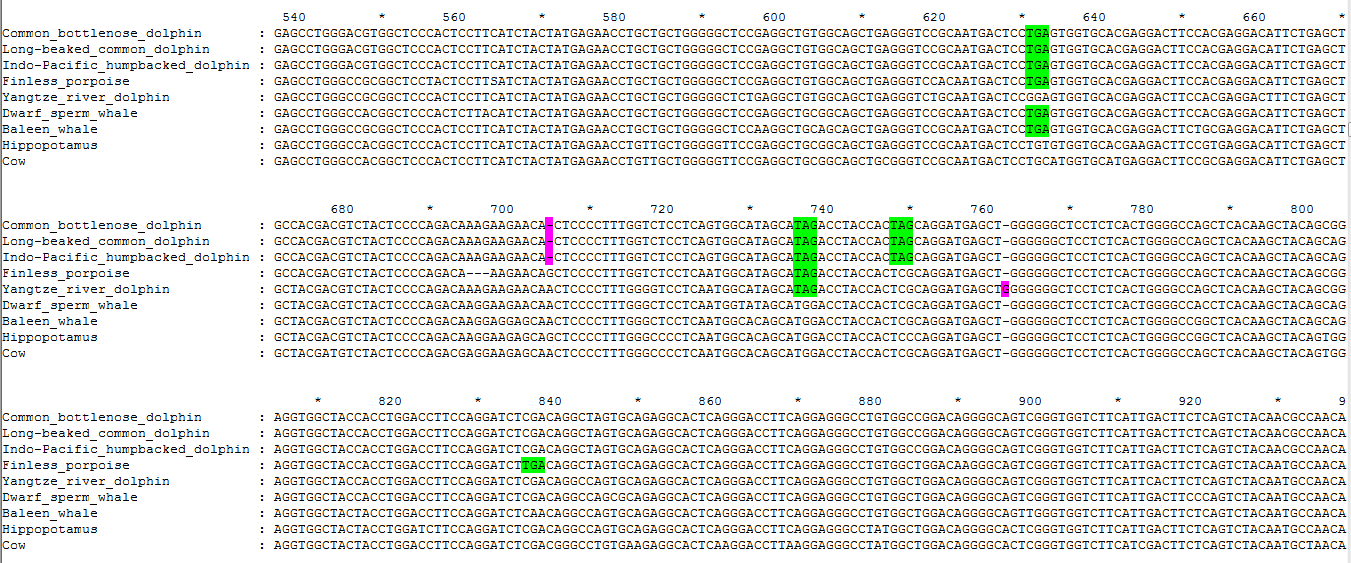

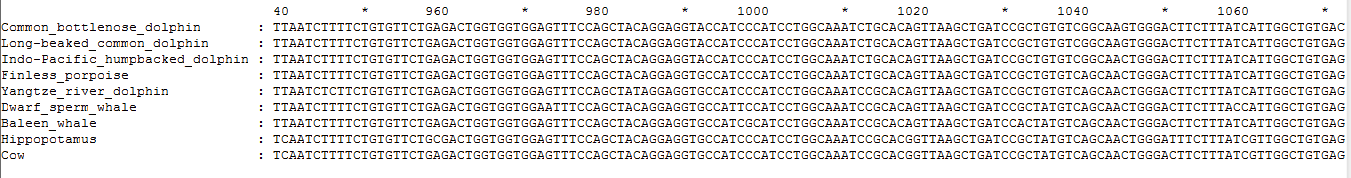


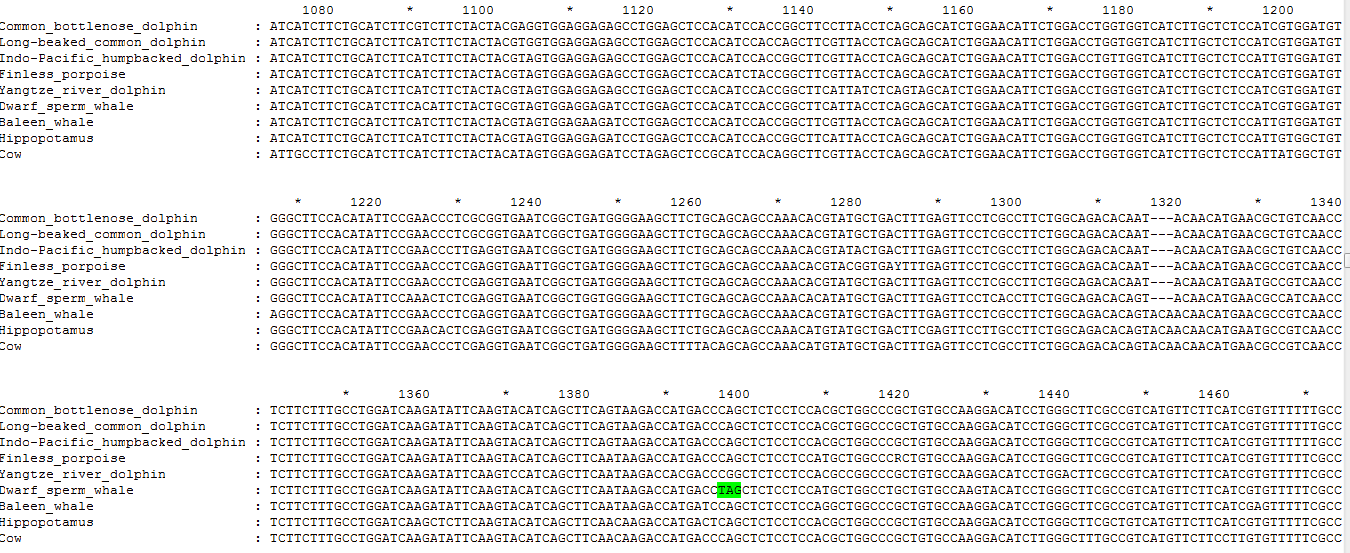

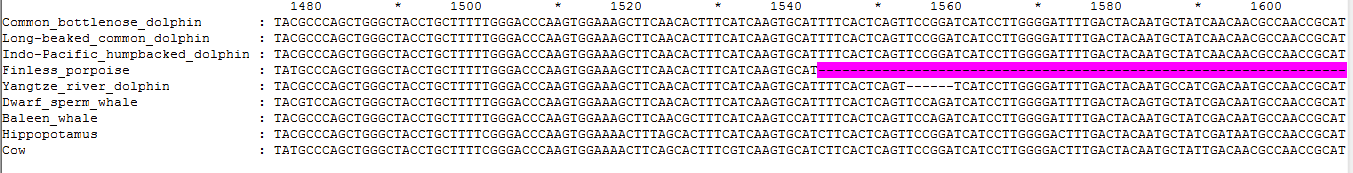


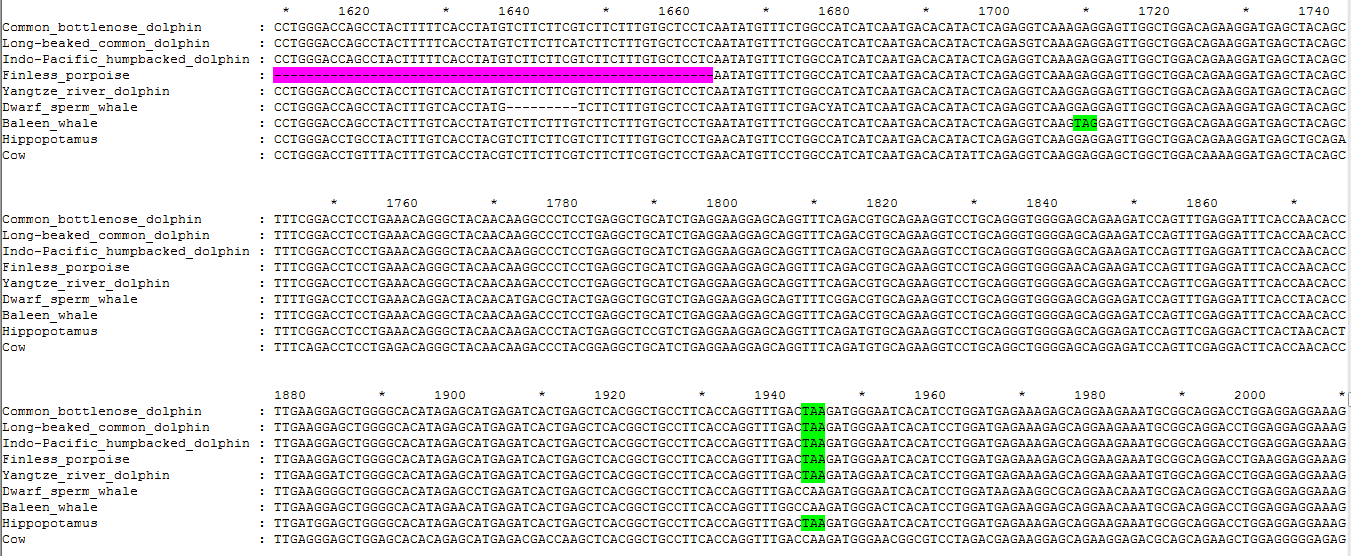

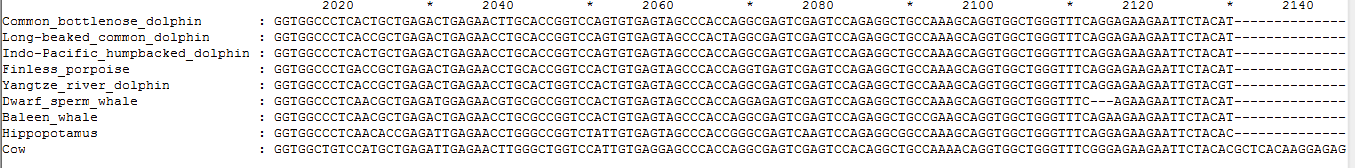


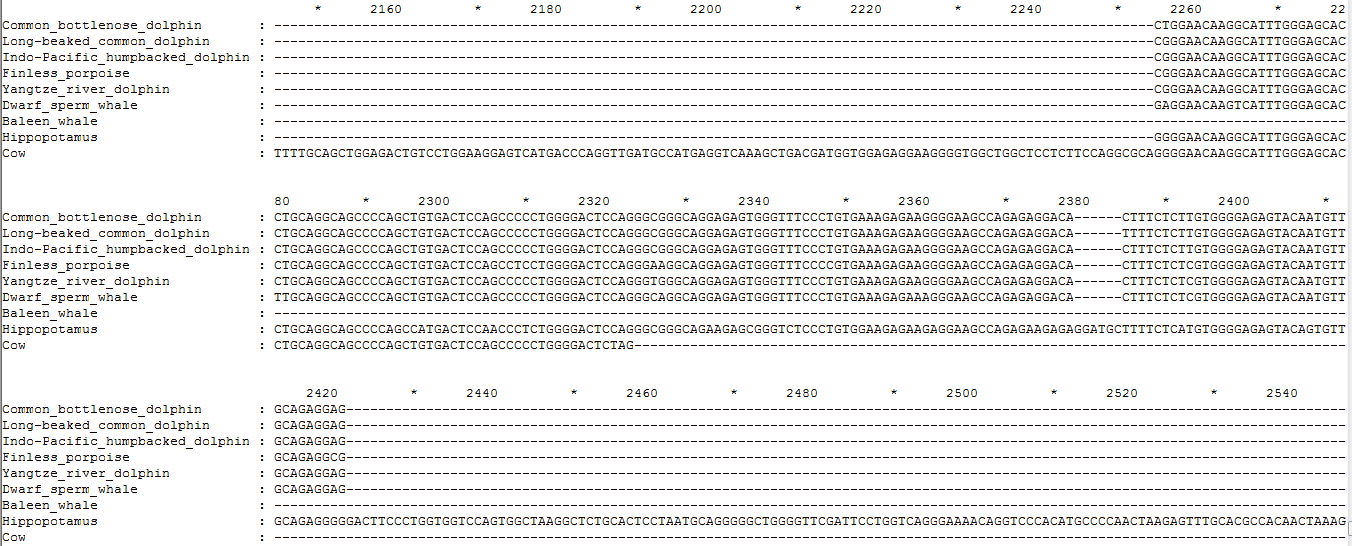

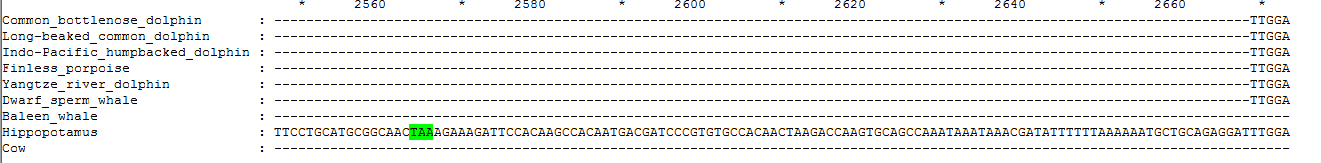


**Fig. S2: Indels and premature stop codons in *Tas1r1*.** Indels were highlighted in red, while premature stop codon were indicated in green.


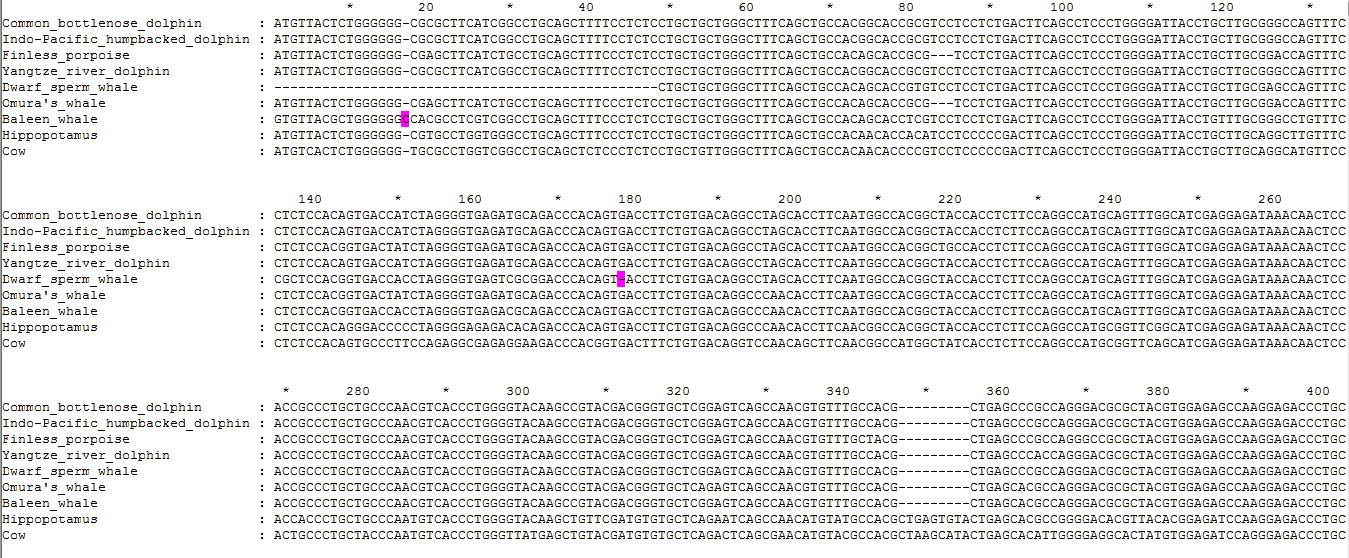

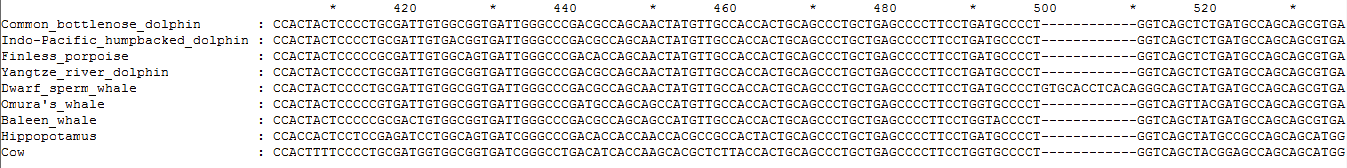


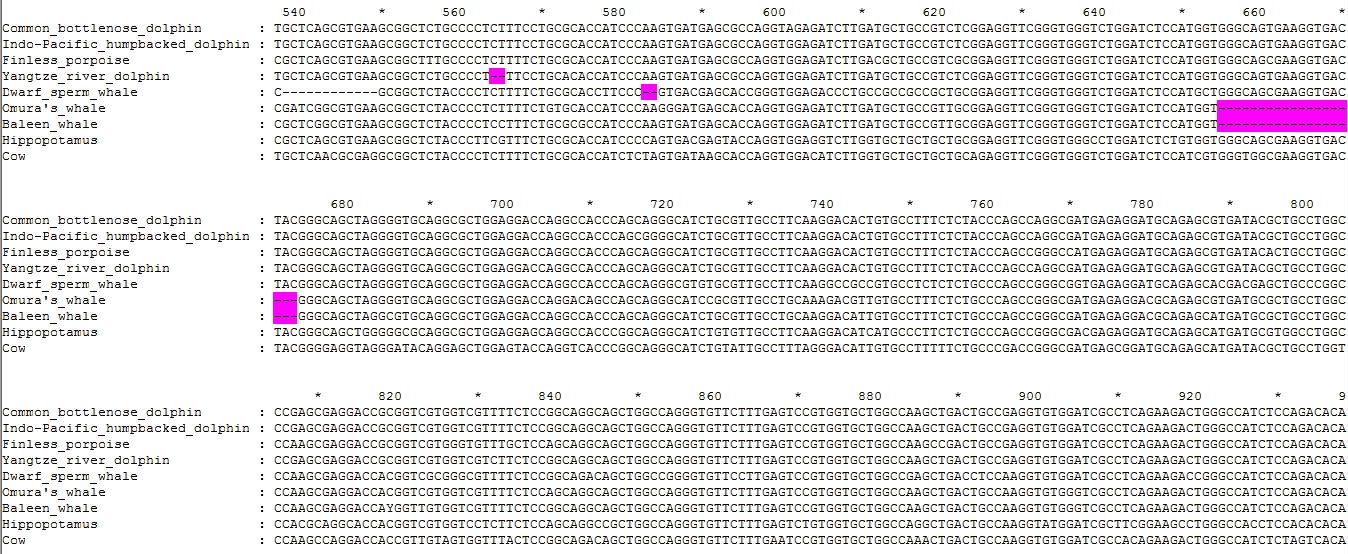

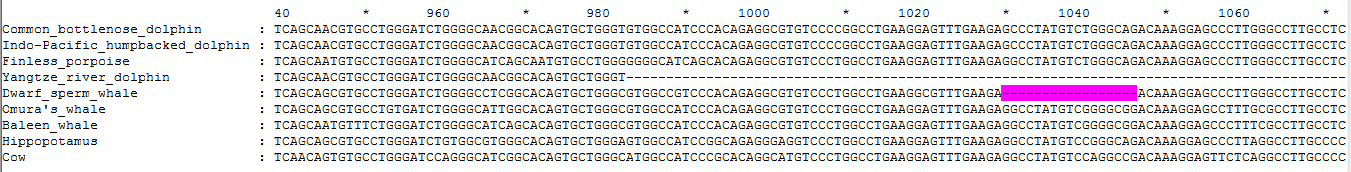


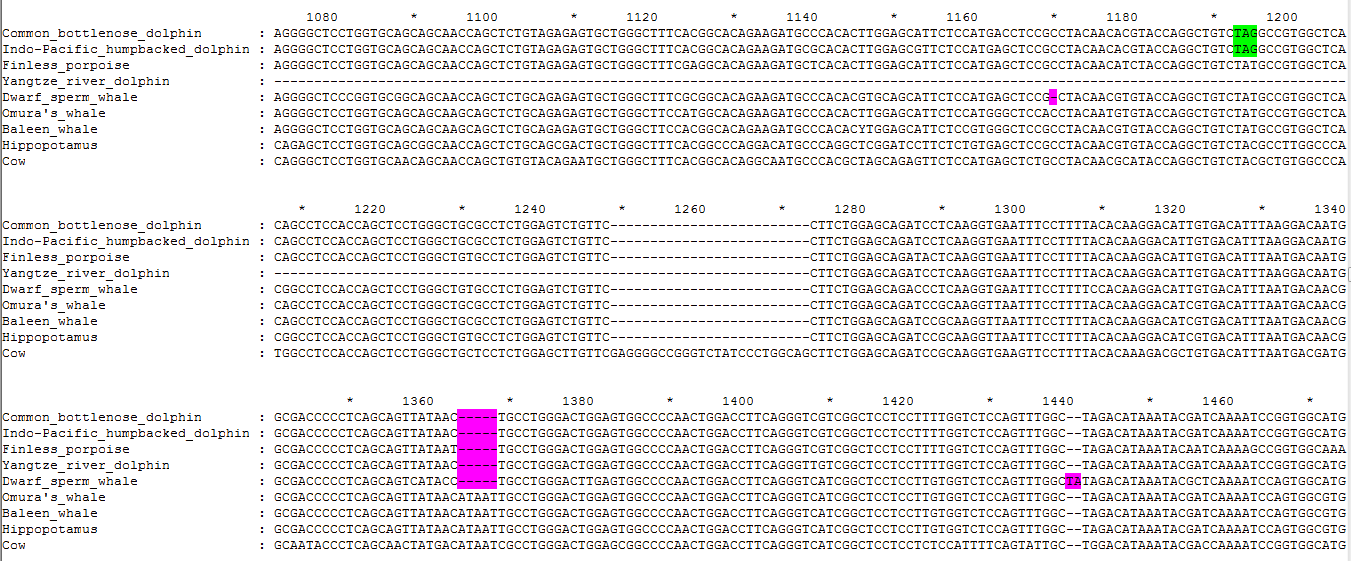

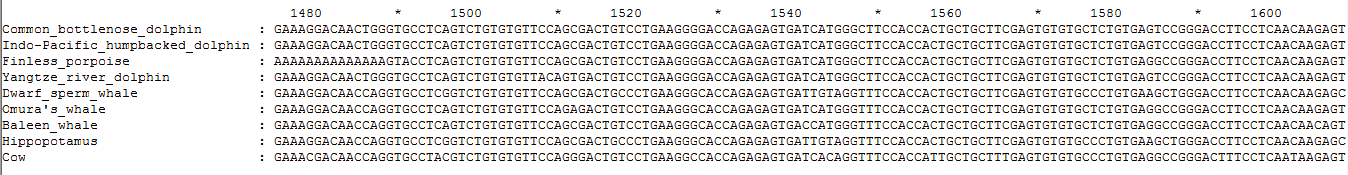


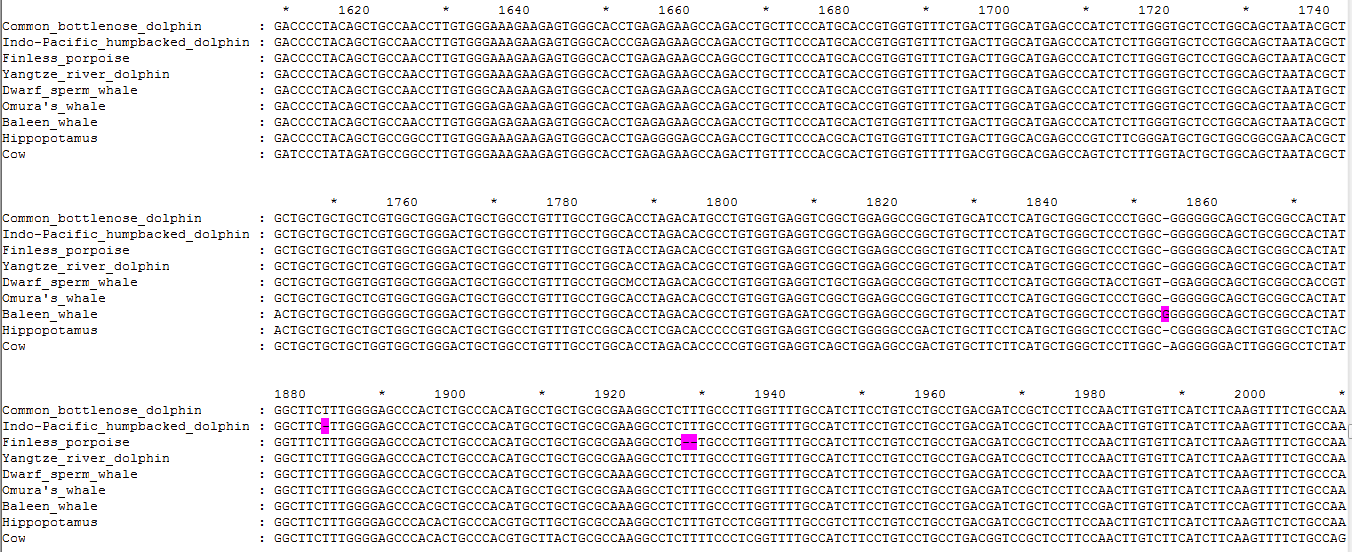

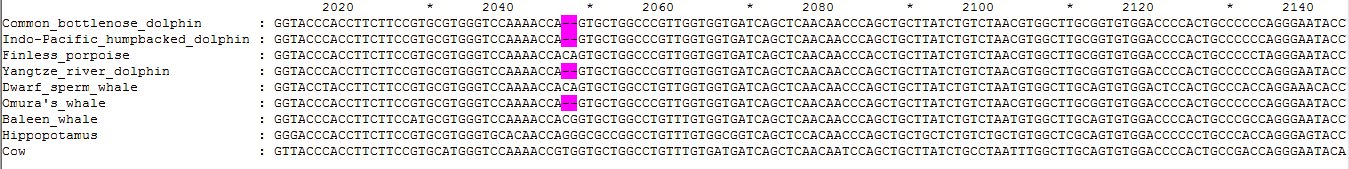


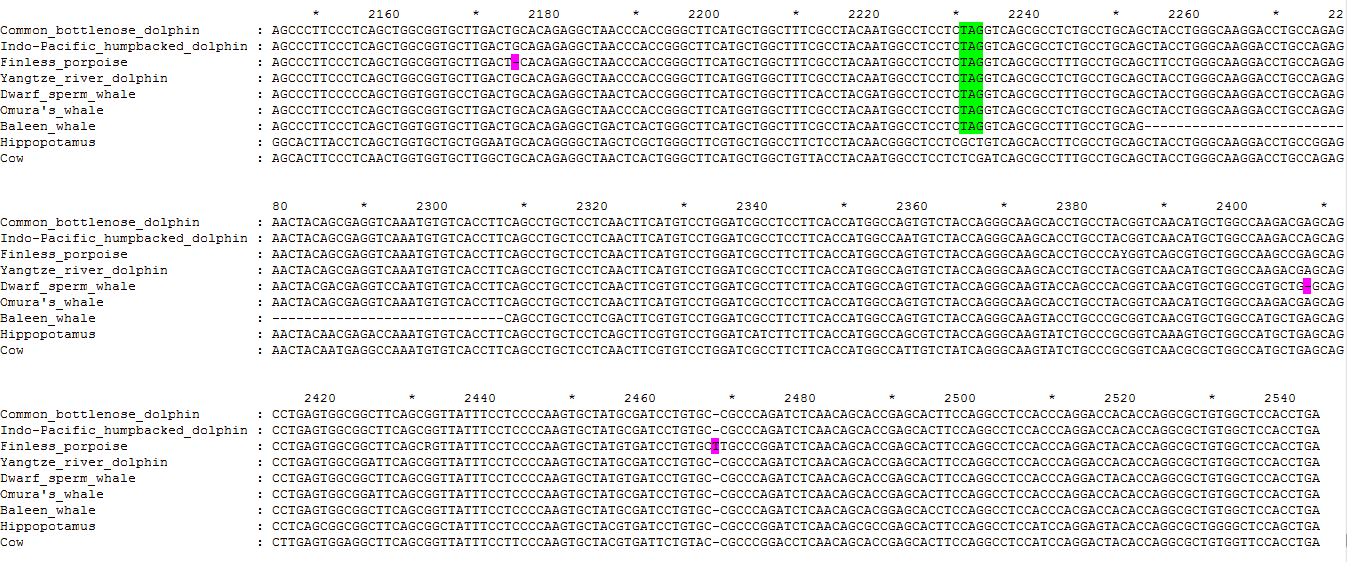


**Fig. S3: Indels and premature stop codons in *Tas1r2*.** Indels were highlighted in red, while premature stop codon were indicated in green.


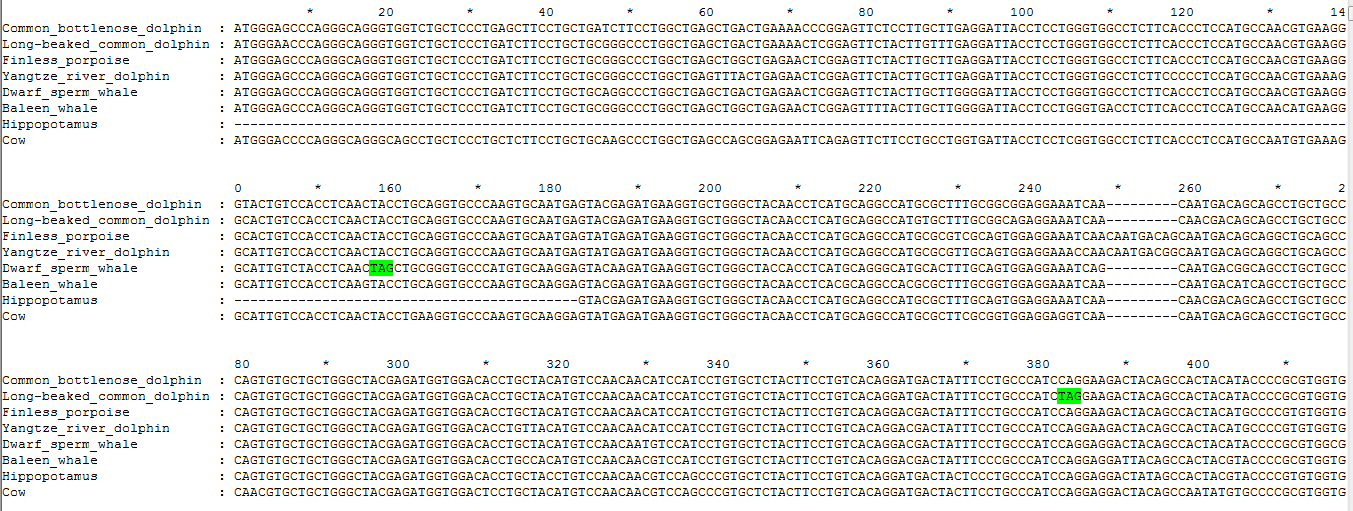

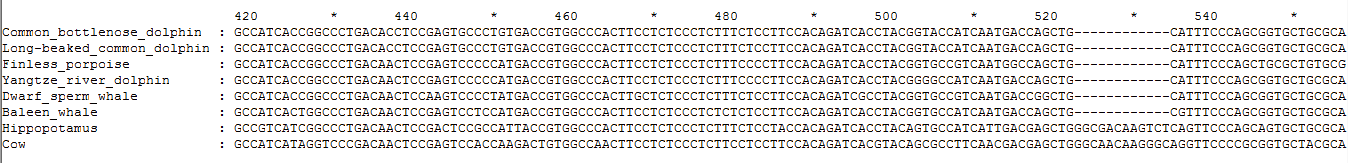


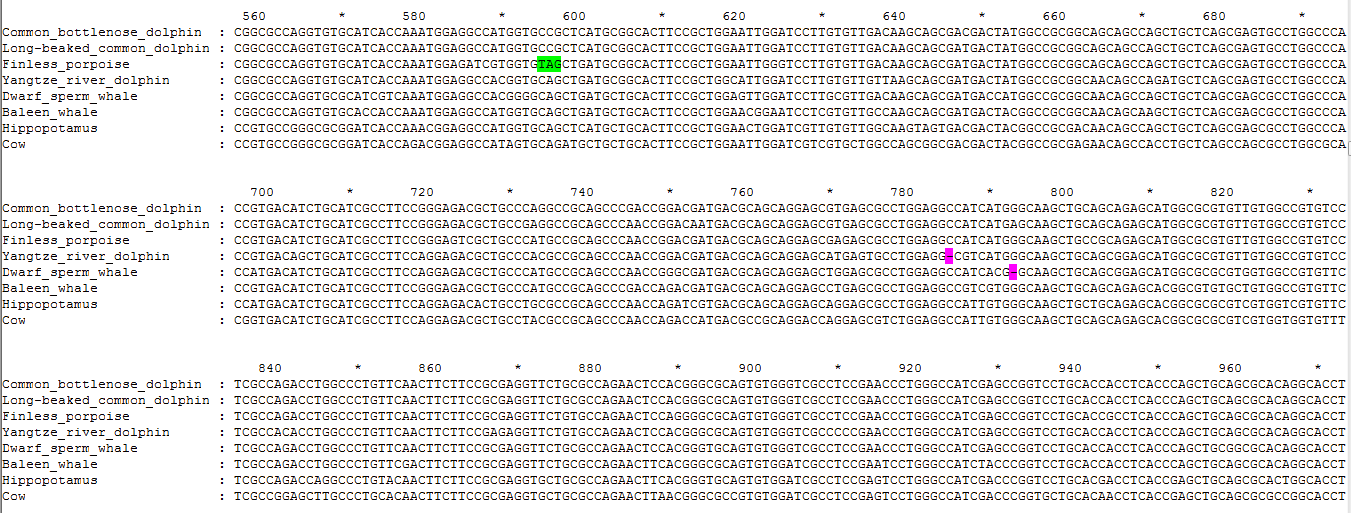

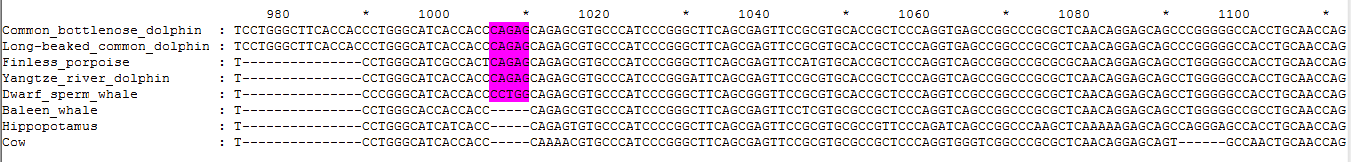

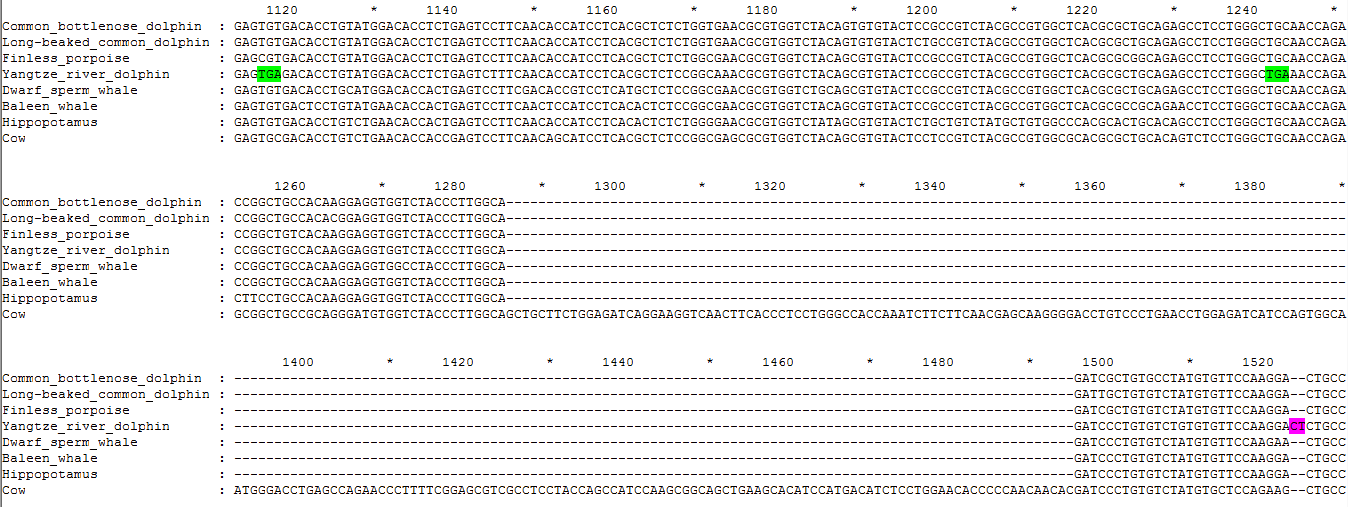

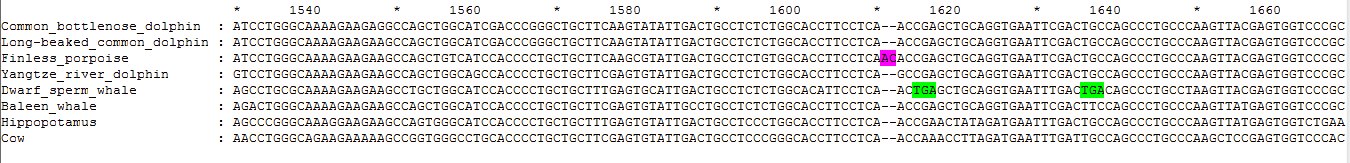

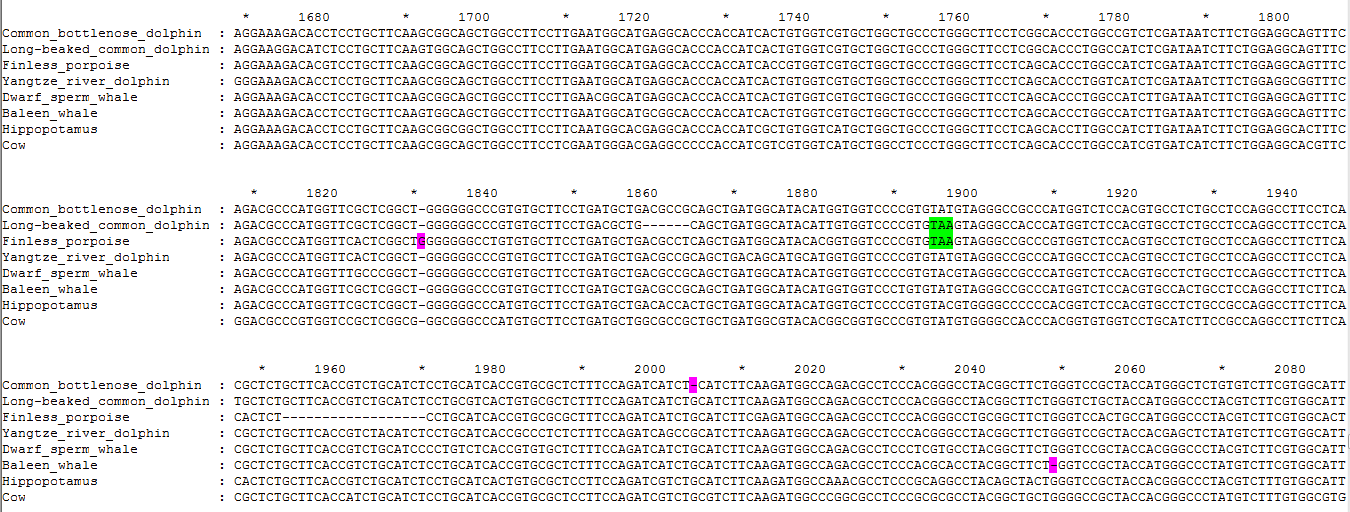

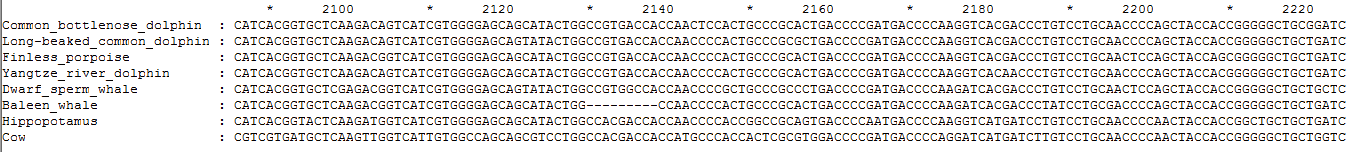

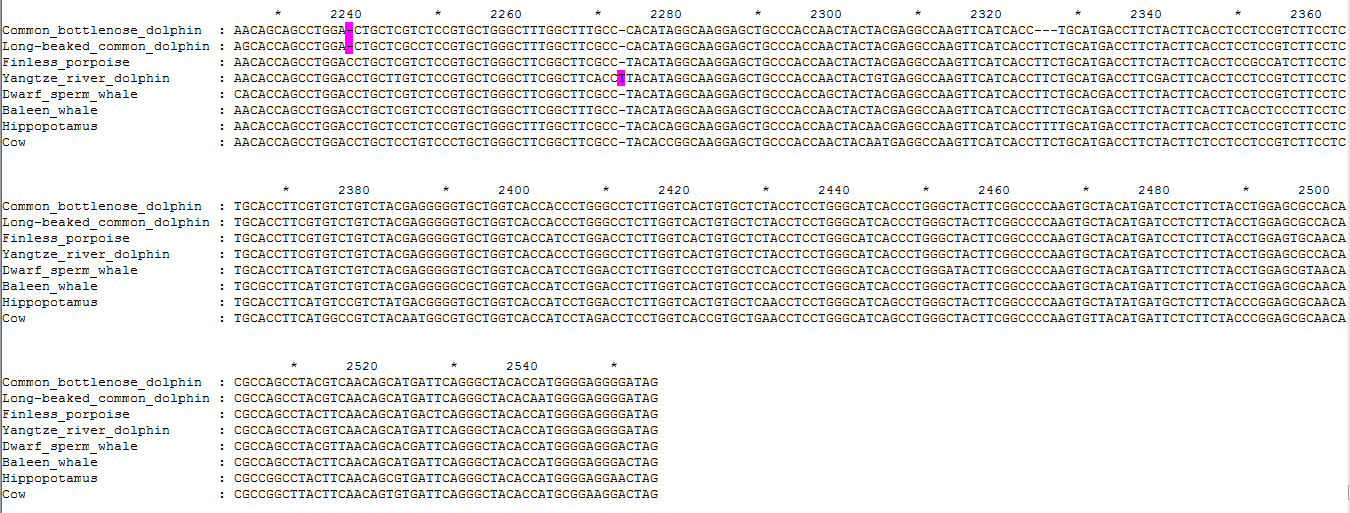


**Fig. S4: Indels and premature stop codons in *Tas2r1*.** Indels were highlighted in red, while premature stop codon were indicated in green.


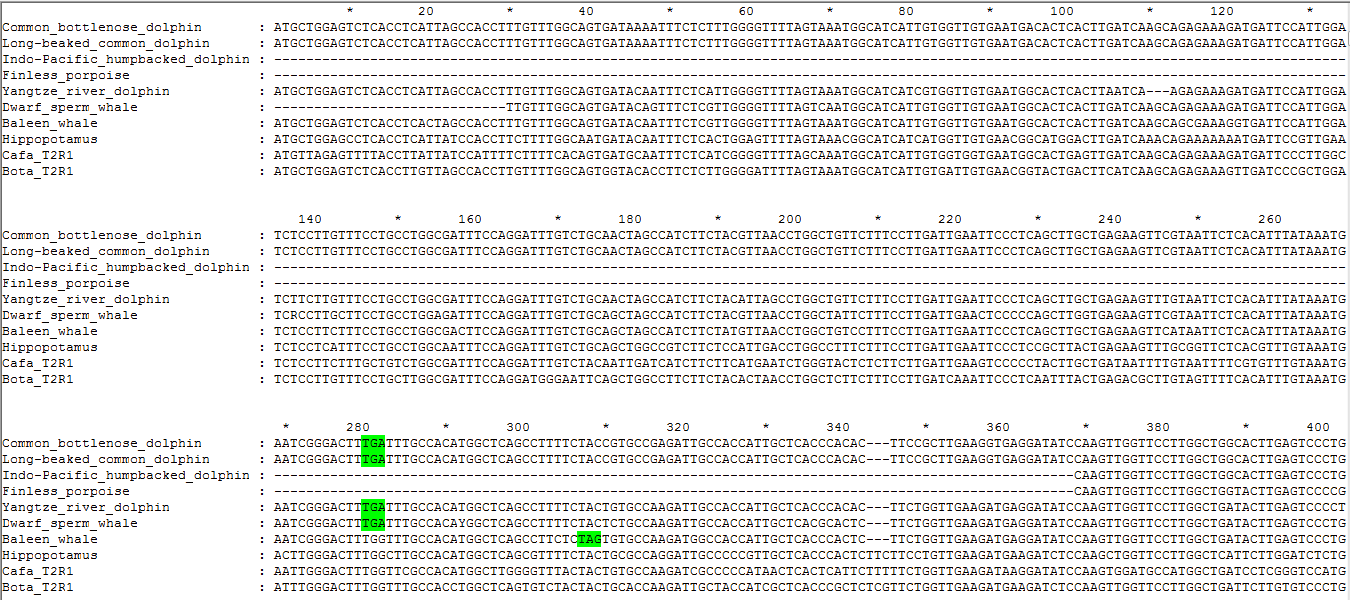

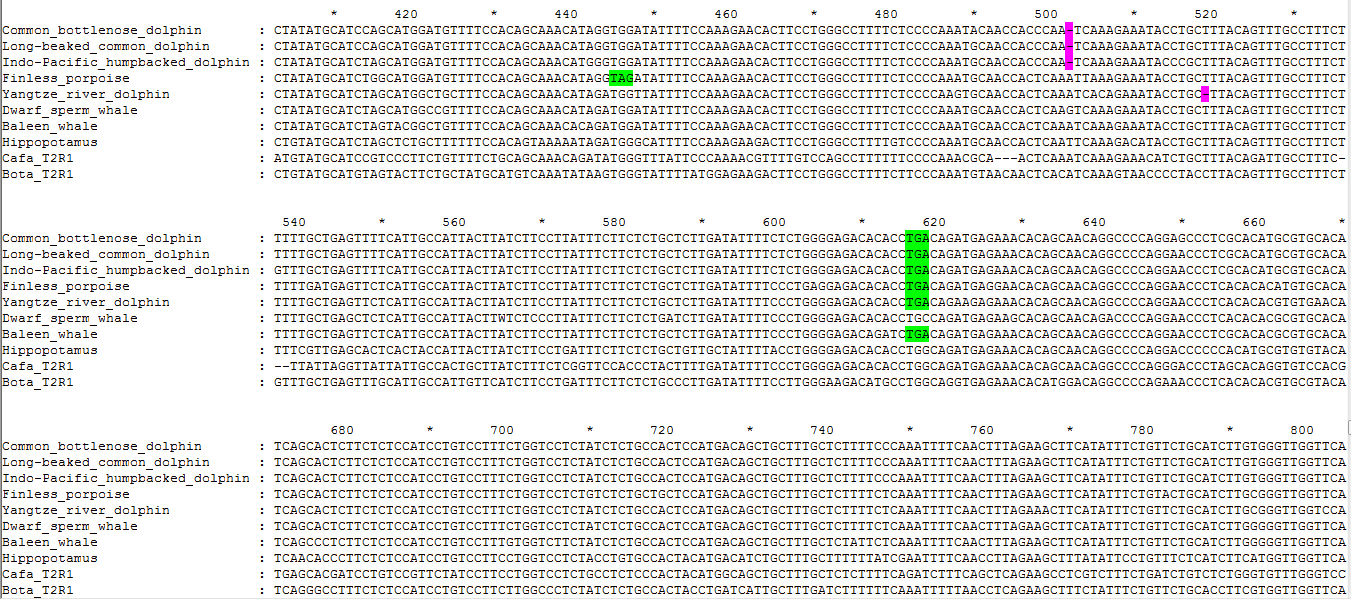

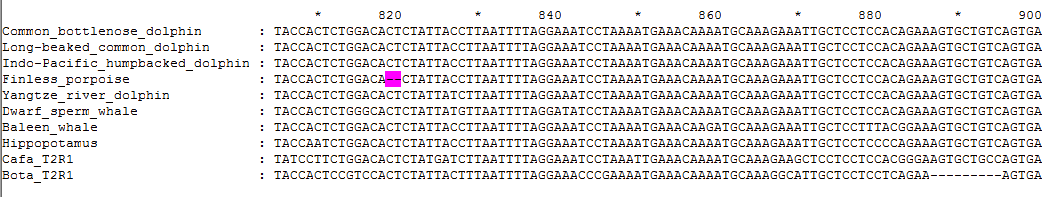


**Fig. S5: Indels and premature stop codons in *Tas2r2*.** Indels were highlighted in red, while premature stop codon were indicated in green.


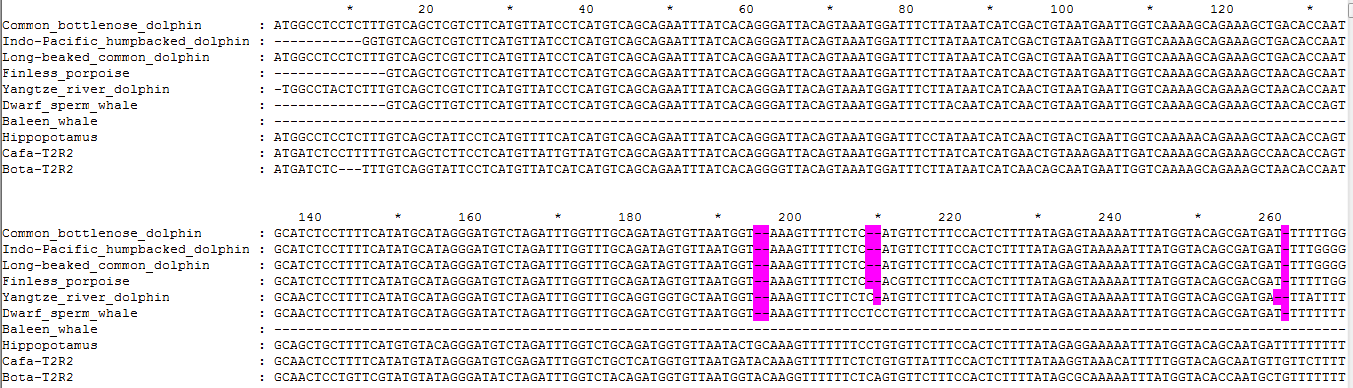


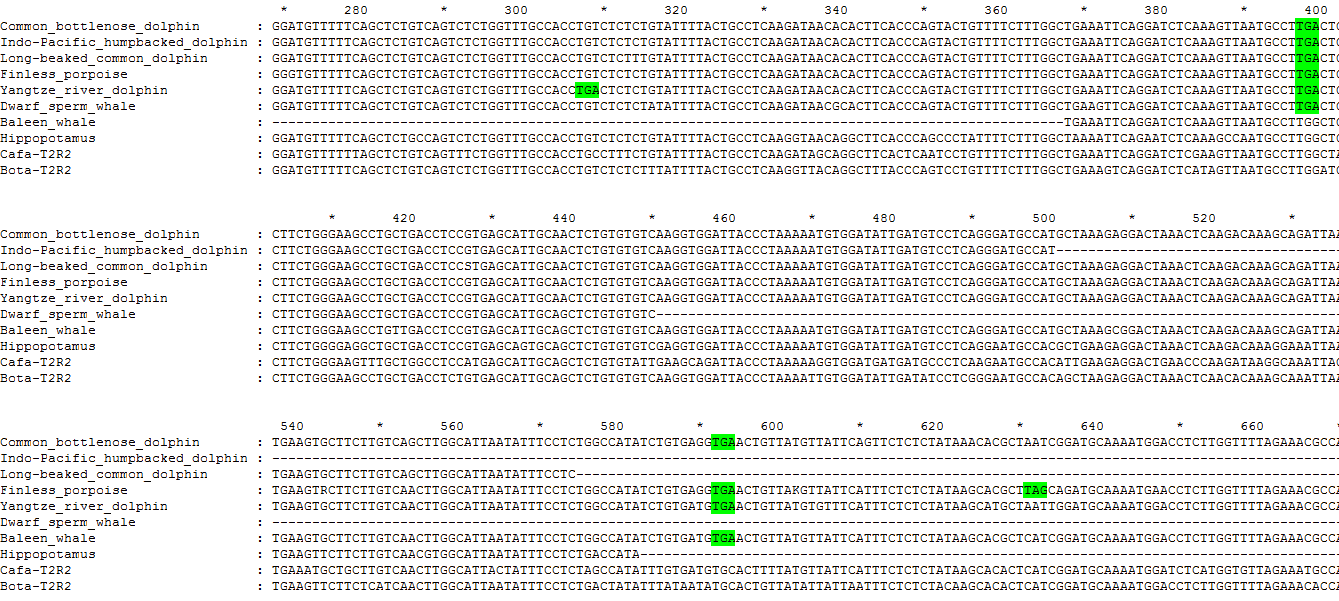

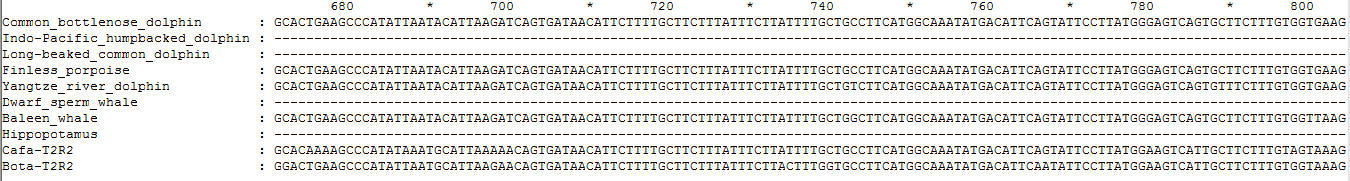

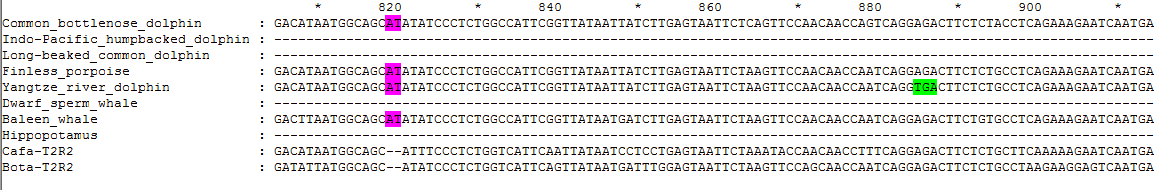


**Fig. S6: Indels and premature stop codons in *Tas2r3*.** Indels were highlighted in red, while premature stop codon were indicated in green.


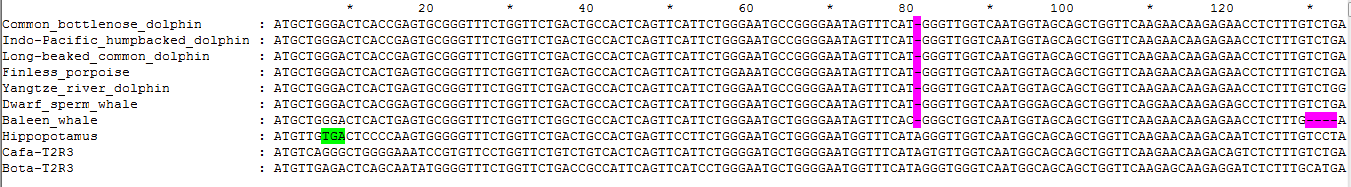

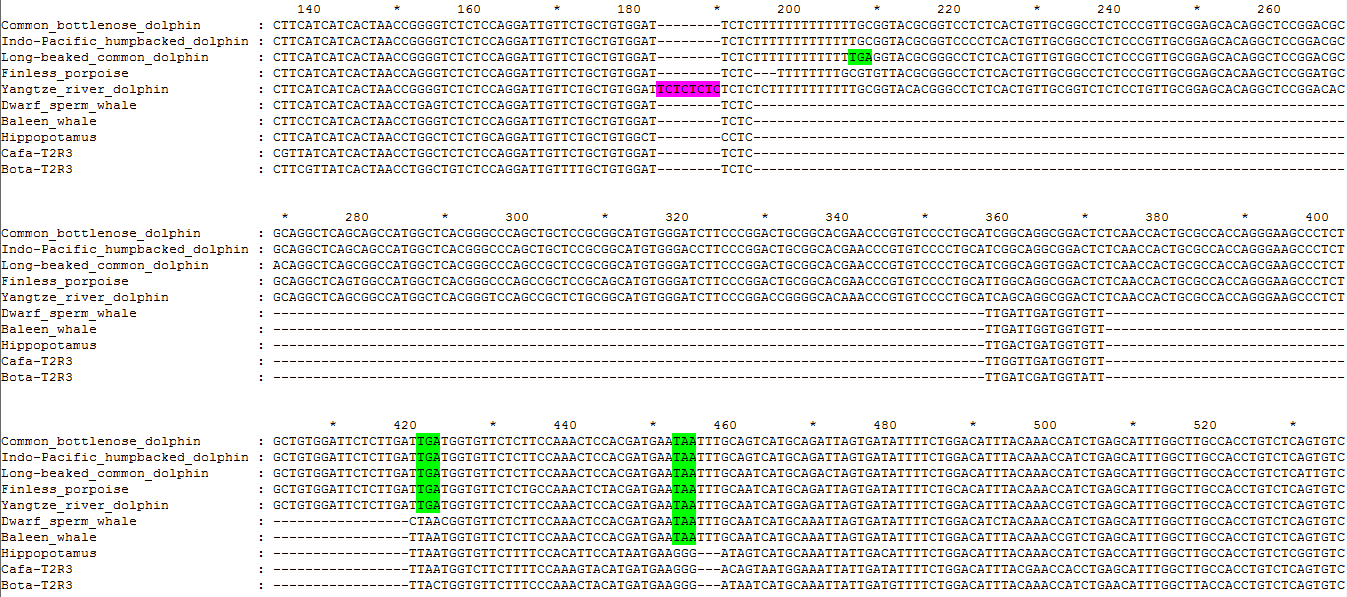

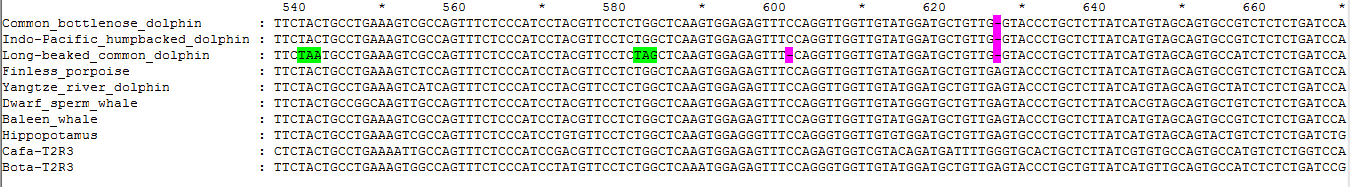

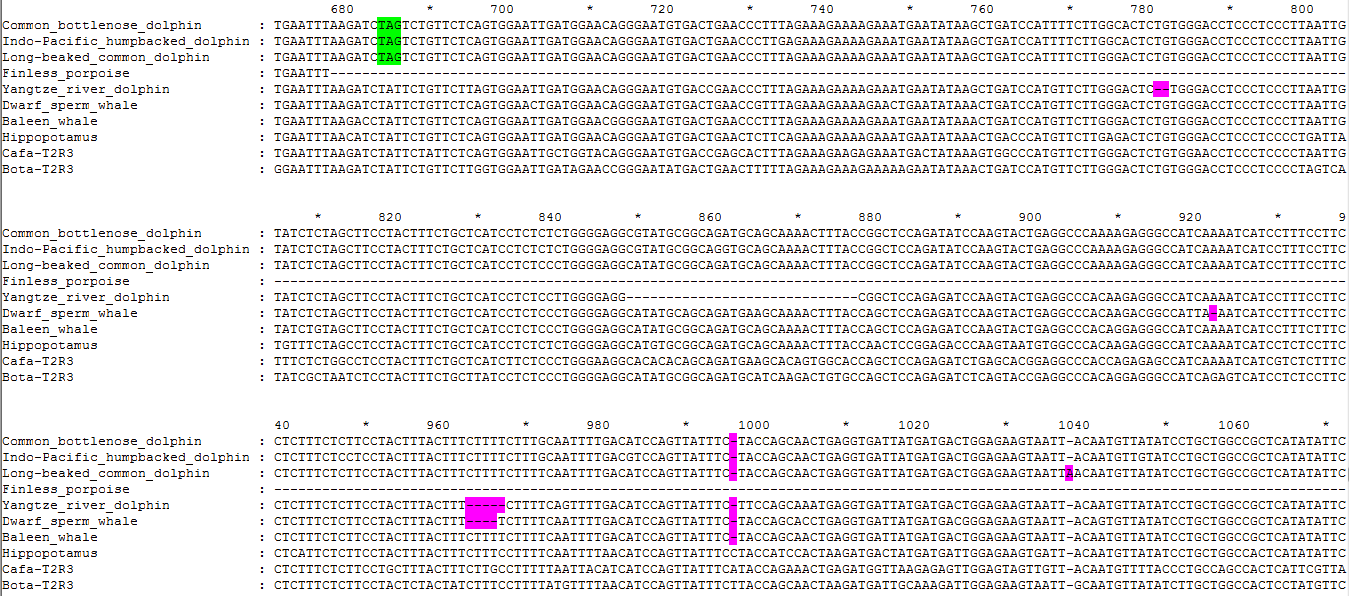

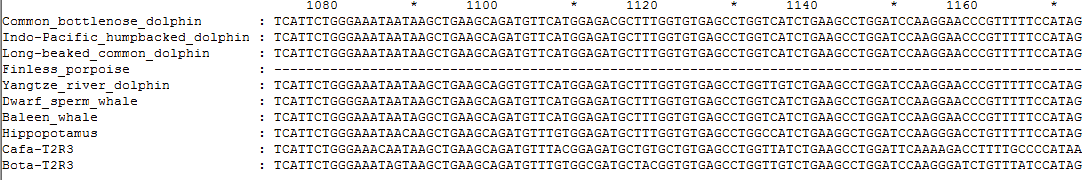


**Fig. S7: Indels and premature stop codons in *Tas2r5*.** Indels were highlighted in red, while premature stop codon were indicated in green.


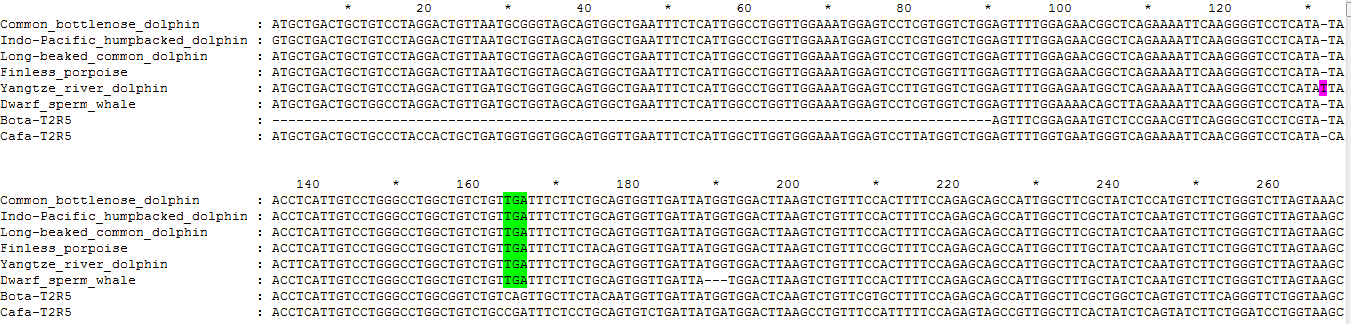

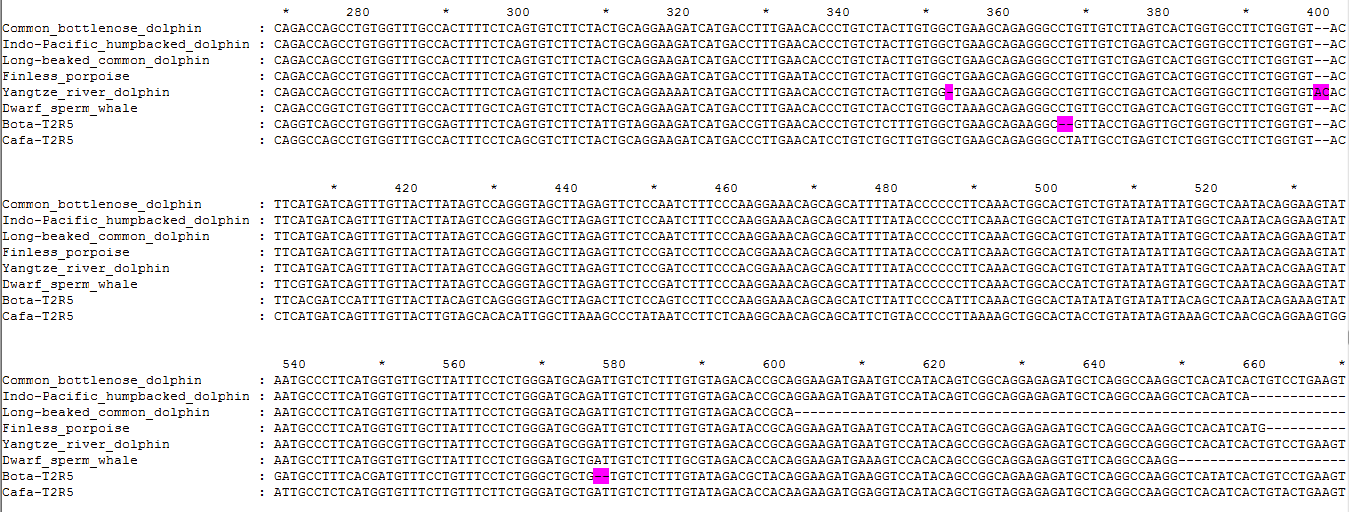

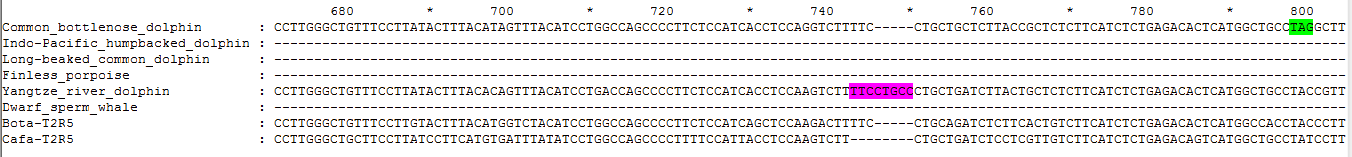

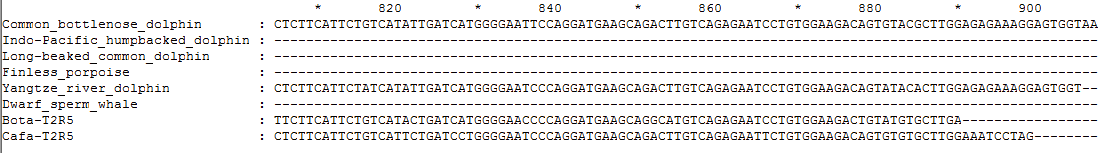


**Fig. S8: Indels and premature stop codons in *Tas2r16.*** Indels were highlighted in red, while premature stop codon were indicated in green.


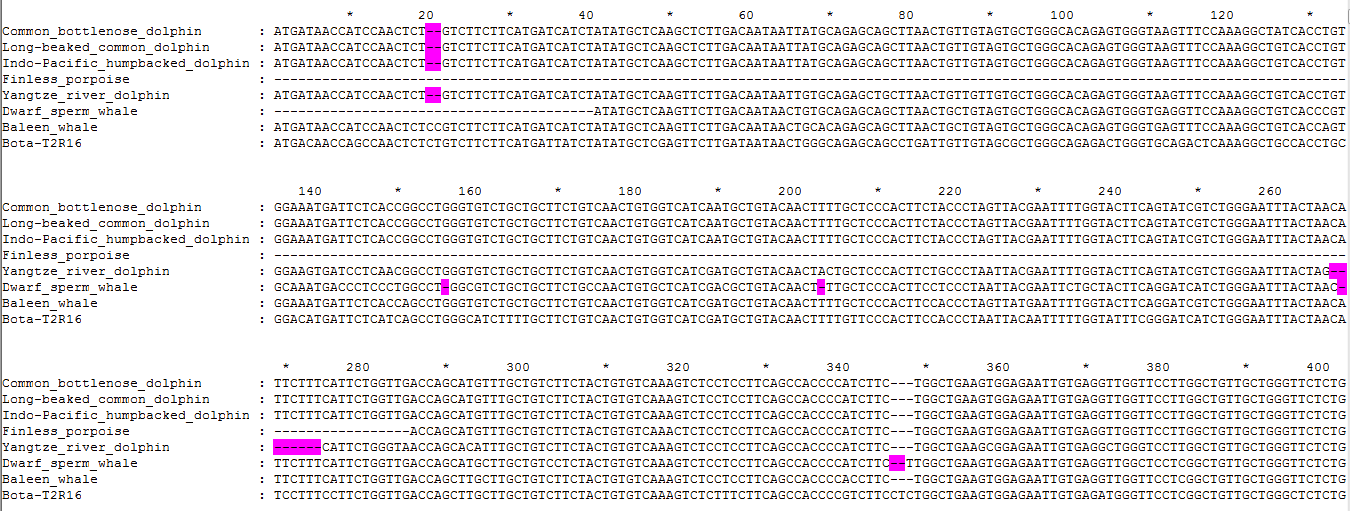

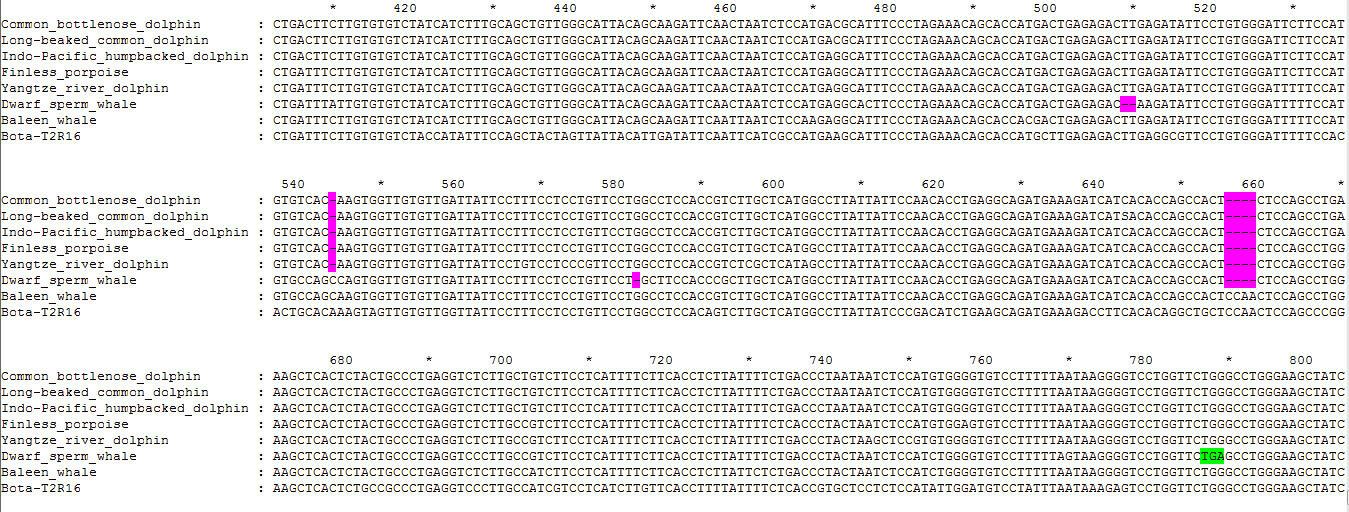

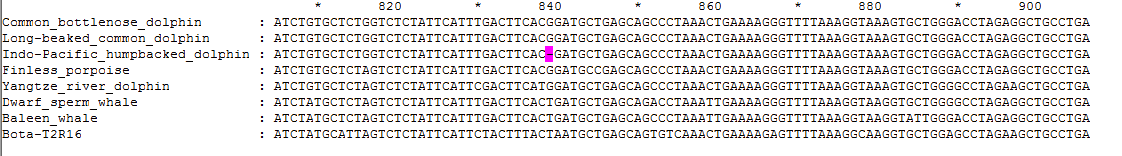


**Fig. S9: Indels and premature stop codons in *Tas2r38.*** Indels were highlighted in red, while premature stop codon were indicated in green.


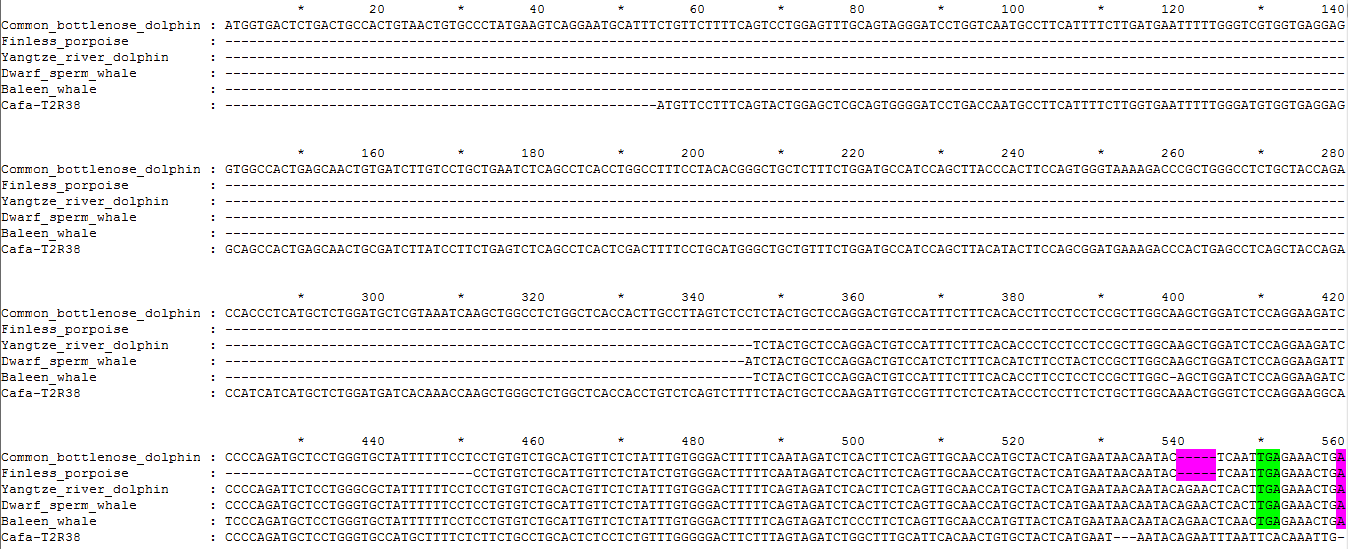

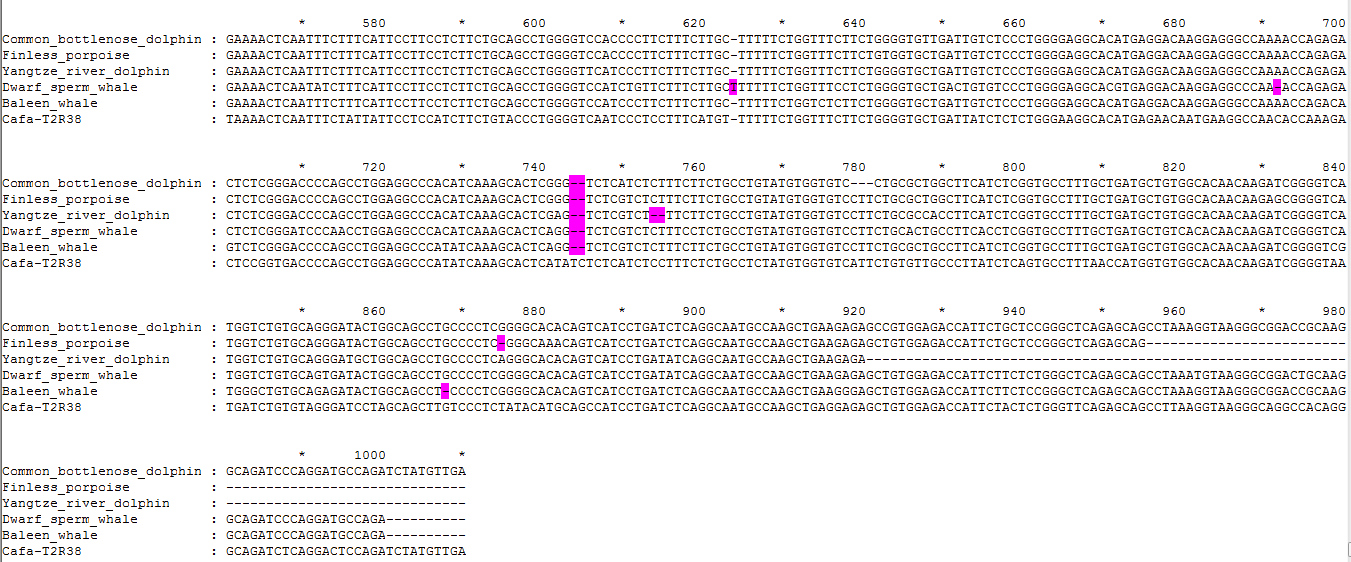


**Fig. S10: Indels and premature stop codons in *Tas2r39.*** Indels were highlighted in red, while premature stop codon were indicated in green.


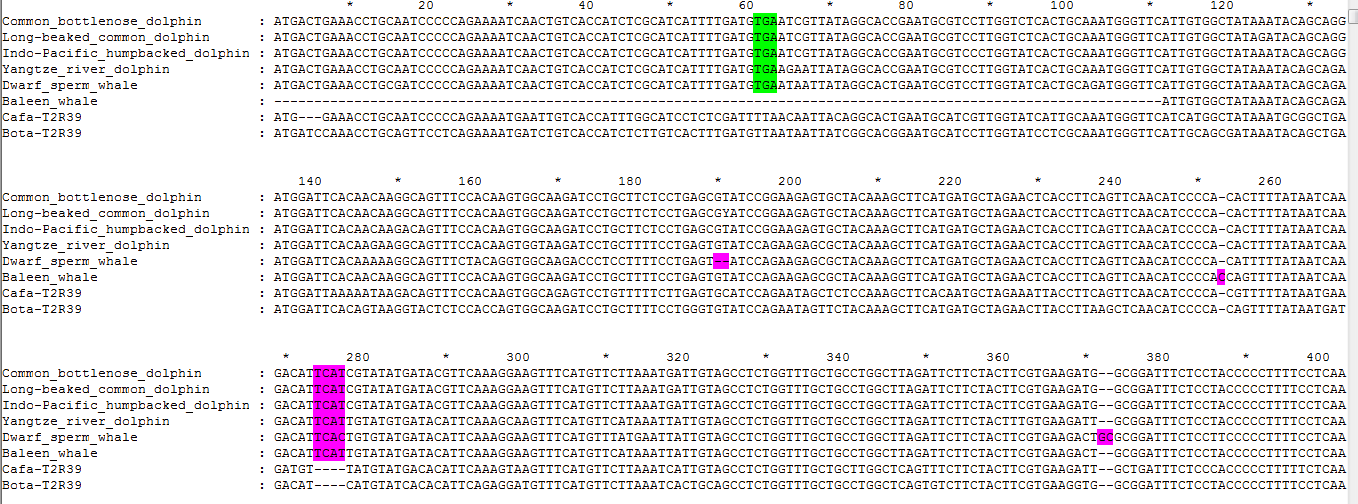

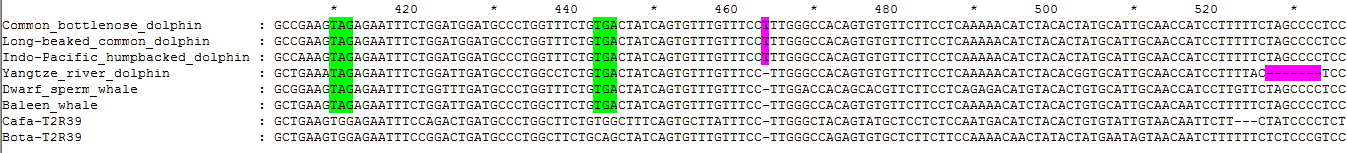


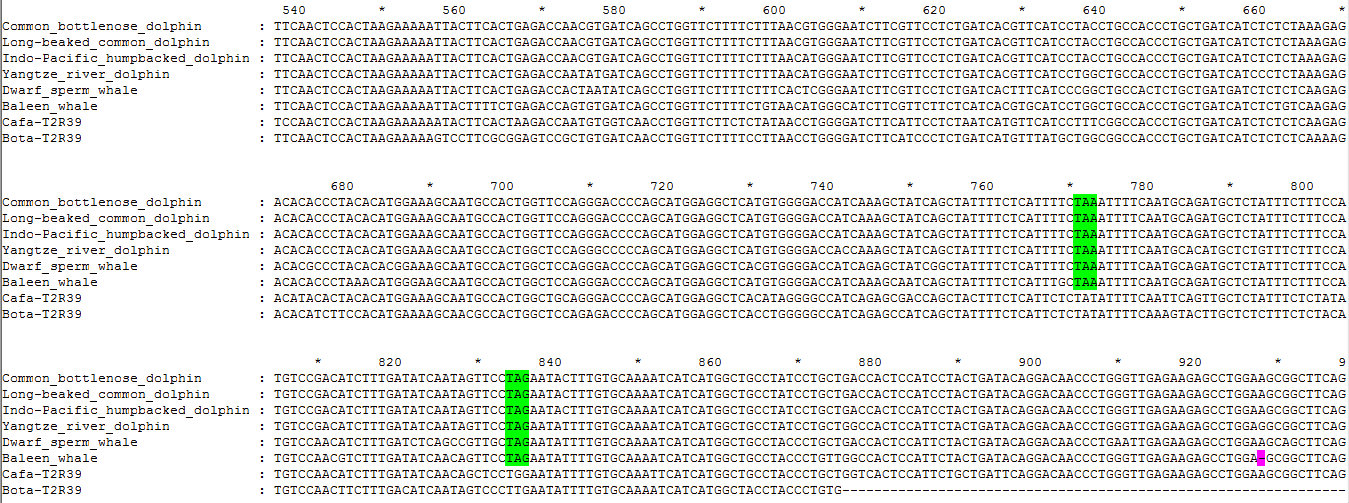

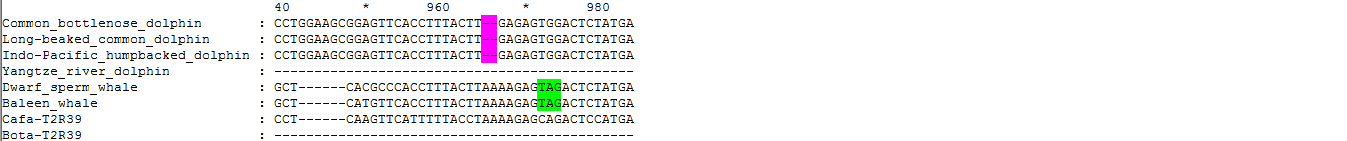


**Fig. S11: Indels and premature stop codons in *Tas2r60.*** Indels were highlighted in red, while premature stop codon were indicated in green.


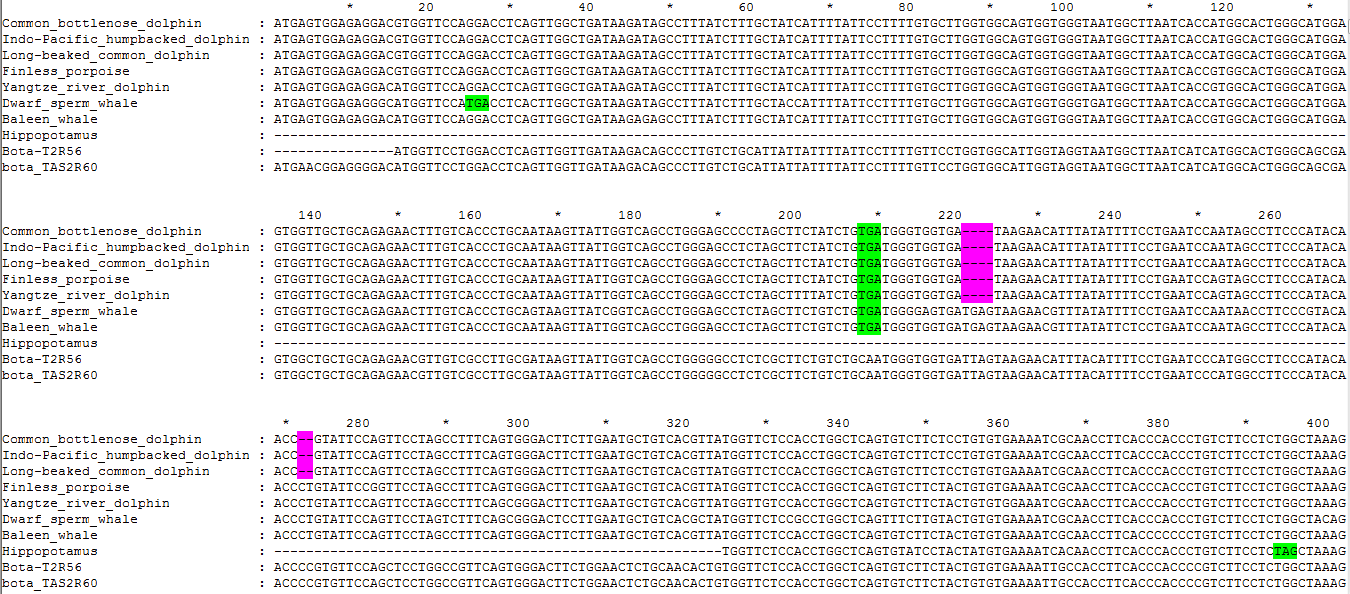

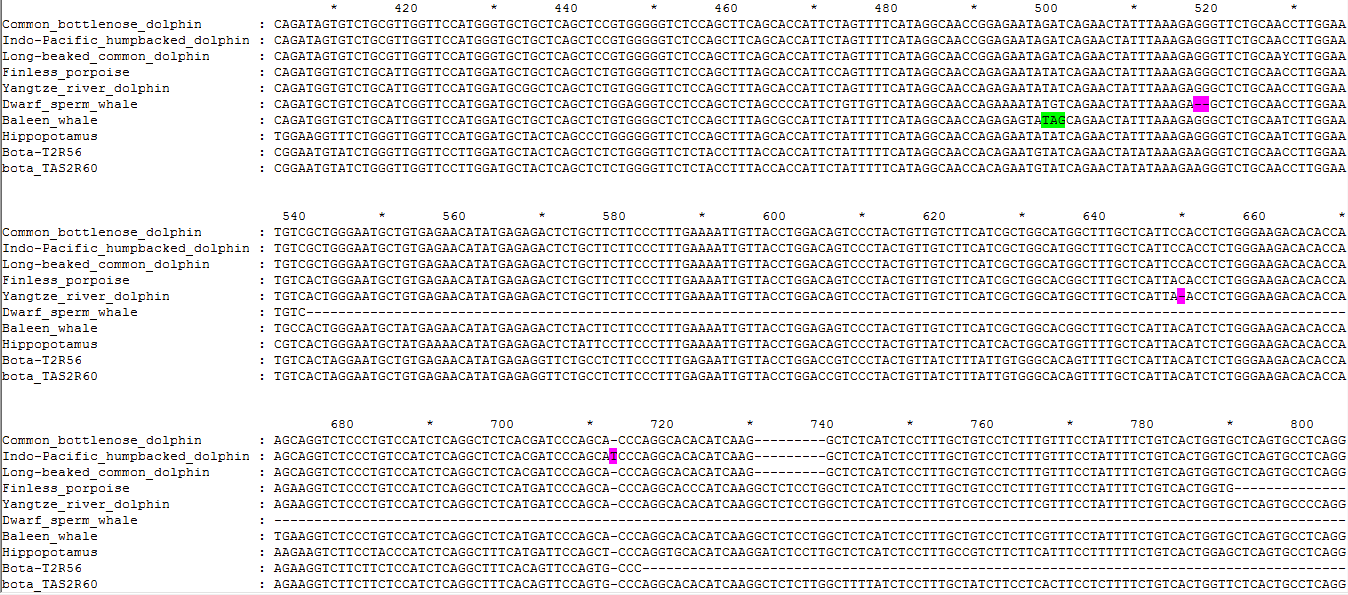

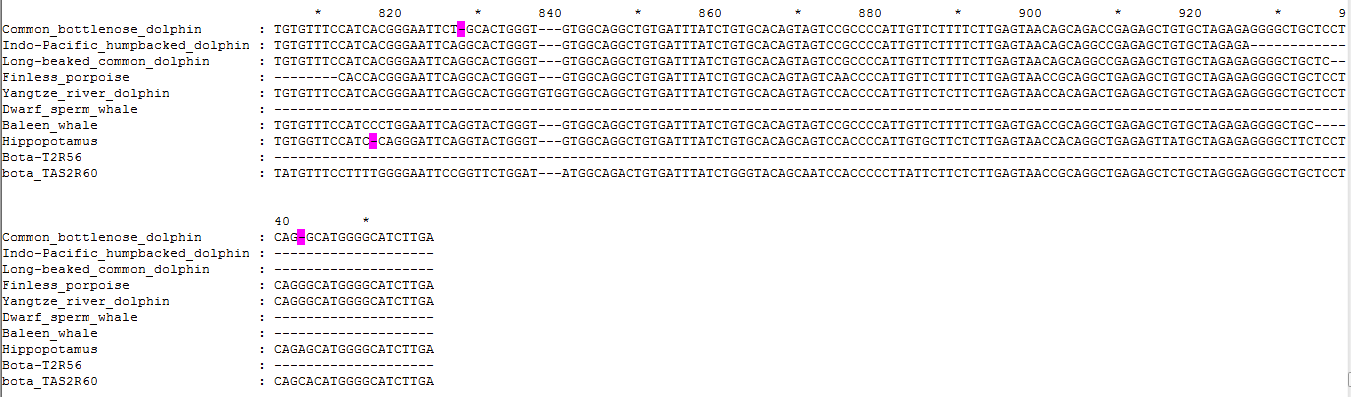

Supplement: Additional file 3: Figures S1-S11. — Indels and premature stop codons in Pkdl21, Tas1r1, Tas1r2, Tas2r1-3, Tas2r5, Tas2r16, Tas2r38-39, and Tas2r60. Indels are highlighted in red, while premature stop codons are indicated in green. [file 12862_2014_218_MOESM3_ESM.doc]
