# Supplementary material for: The loss of taste genes in cetaceans
Source: BMC Evol Biol. 2014 Oct 12;14:218. doi: 10.1186/s12862-014-0218-8 (PMC4232718; doi:10.1186/s12862-014-0218-8)
Supplement: Additional file 4: Table S8. — The location of the first premature stop codon in each pseudogenized taste receptor gene. N represents the N-terminus, FEL represents the First extracellular loop, SEL represents Second extracellular loop, TEL represents the Third extracellular loop, FIL represents the Fourth intracellular loop, TM represents transmembrane domain, SIL represents Second intracellular loop, and NA represents non-amplification in our analysis. [file 12862_2014_218_MOESM4_ESM.doc]

**Table S8. The location of the first premature stop coden in each pseudogenized taste receptor gene.**

The location of the first premature stop coden in each individual of the five taste receptor genes, and the domains after this first premature stop coden were not represented. Note: N represents the N-terminus, FEL represents the First extracellular loop, SEL represents Second extracellular loop, TEL represents the Third extracellular loop, FIL represents the Fourth intracellular loop, TM represents transmembrane domain, SIL represents Second intracellular loop, and NA represents non-amplification in our analysis.

|  | *Tas1r1* | *Tas1r2* | *Pkd2l1* | *Tas2r1* | *Tas2r2* | *Tas2r3* | *Tas2r5* | *Tas2r16* | *Tas2r38* | *Tas2r39* | *Tas2r60* |
| --- | --- | --- | --- | --- | --- | --- | --- | --- | --- | --- | --- |
| *Tursiops truncatus* | N | N | FEL | Third TM | SEL | Fourth TM | Second TM | SIL | TEL | First TM | FIL |
| *Delphinus capensis* | N | N | FEL | Third TM | SEL | Fourth TM | Second TM | SIL | NA | First TM | FIL |
| *Sousa chinensis* | N | NA | FEL | Fifth TM | SEL | Fourth TM | Second TM | SIL | NA | First TM | FIL |
| *Neophocaena phocaenoides* | N | N | FEL | Third TM | SEL | Fourth TM | Second TM | Sixth TM | TEL | NA | FIL |
| *Lipotes vexillifer* | N | N | FEL | Third TM | TEL | TEL | Second TM | SIL | TEL | First TM | FIL |
| *Kogia sima* | N | N | SEL | Third TM | SEL | FIL | Second TM | SIL | TEL | First TM | FIL |
| *Banaenoptera omurai* | N | NA | NA | NA | NA | NA | NA | NA | NA | NA | NA |
| *Balaenoptera acutorostrata* | N | N | FIL | Third TM | Fifth  TM | FIL | NA | Intact | TEL | FIL | FIL |
| *Hippopotamus amphibious* | Intact | Intact | FIL | Intact | Presumed intact | FEL | NA | NA | NA | NA | Third TM |
