# Supplementary material for: The loss of taste genes in cetaceans
Source: BMC Evol Biol. 2014 Oct 12;14:218. doi: 10.1186/s12862-014-0218-8 (PMC4232718; doi:10.1186/s12862-014-0218-8)

**Fig. S12-S22 Indels and premature stop codons mapped on the species tree for pseudogenized taste receptor genes.**

A list of phylogenetic trees of the taste receptor genes analyzed in the present study with all indels and premature stop codons mapped in order. All indels are characterized with bars, with each color representing a different indel and the same color representing the same indel across the tree. Premature stop codons are characterized with ellipses; we did not differentiate premature stop codons and all have the same color.


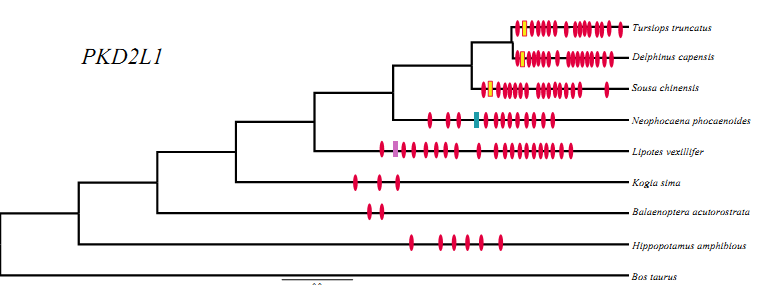


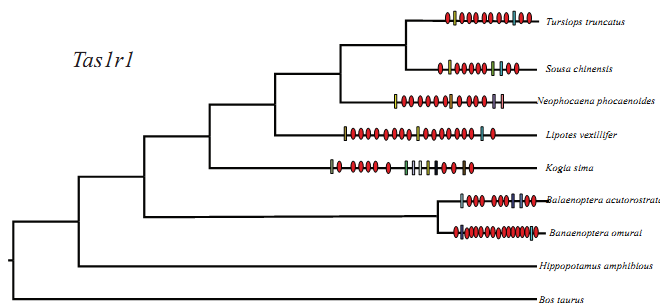


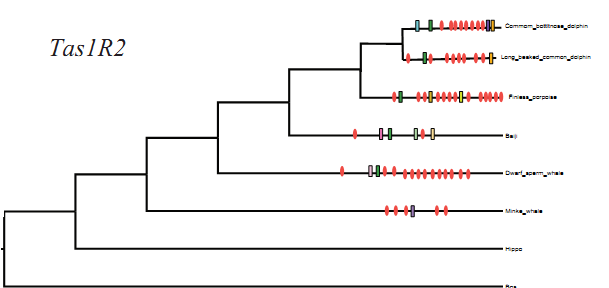


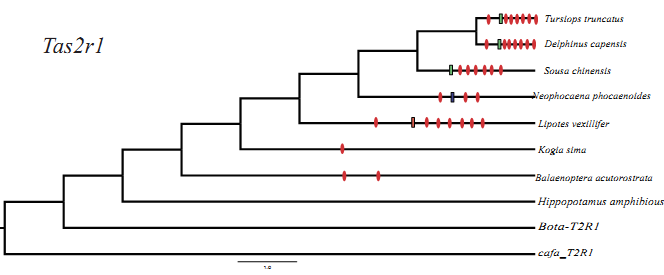


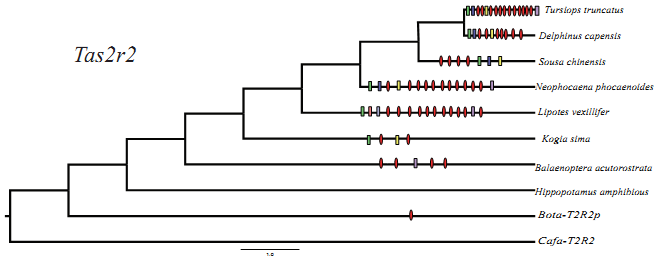


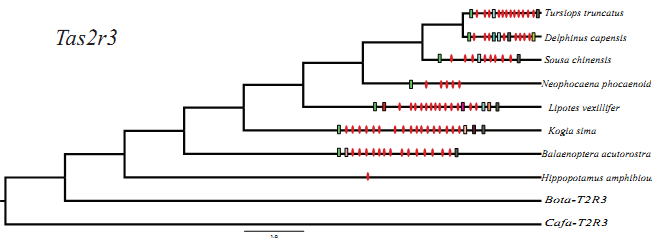


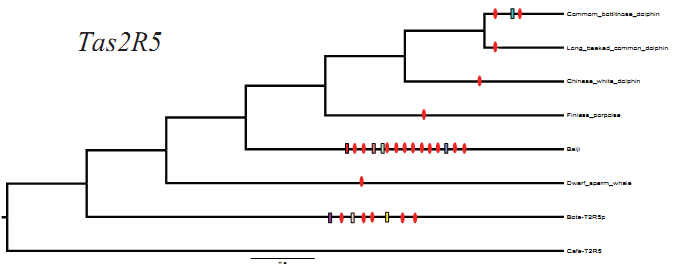


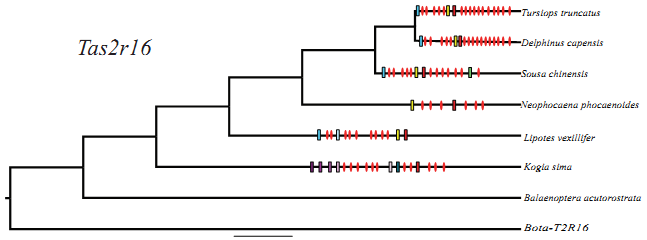


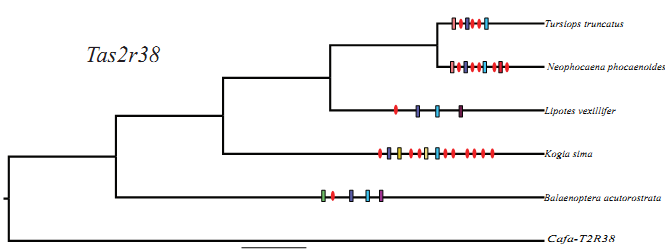


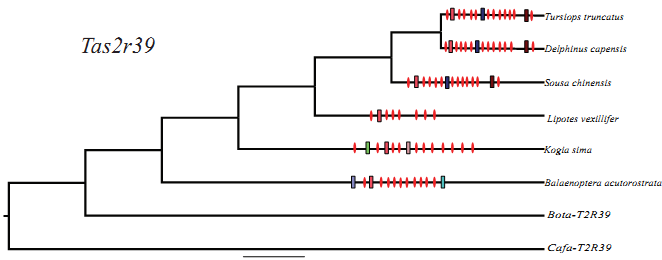


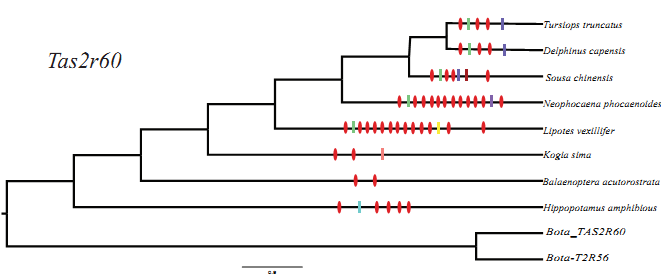

Supplement: Additional file 5: Figures S12-S22. — Indels and premature stop codons mapped on the species tree for pseudogenized taste receptor genes. A list of phylogenetic trees of the taste receptor genes analyzed in the present study with all indels and premature stop codons mapped in order. All indels are characterized with bars, with each color representing a different indel and the same color representing the same indel across the tree. Premature stop codons are characterized with ellipses; we did not differentiate premature stop codons and all have the same color. [file 12862_2014_218_MOESM5_ESM.doc]
