# Supplementary material for: The loss of taste genes in cetaceans
Source: BMC Evol Biol. 2014 Oct 12;14:218. doi: 10.1186/s12862-014-0218-8 (PMC4232718; doi:10.1186/s12862-014-0218-8)
Supplement: Additional file 6: Tables S9-S15. — Likelihood ratio tests of various models on the selective pressures on seven bitter taste receptor genes. [file 12862_2014_218_MOESM6_ESM.doc]

**Tables S9-S15 Likelihood ratio tests of various models on the selective pressures on seven bitter taste receptor genes.**

***Tas2r1***

| Models | ω | -lnL | np | Models compared | 2Δ (ln L) | p-value |
| --- | --- | --- | --- | --- | --- | --- |
| All branches have one ω(A) | 0.77979 | 3219.62 | 20 |  |  |  |
| All branches have one ω=1(B) | 1 | 3222.26 | 19 | B vs. A | 5.28 | 0.02 |
| The branches with pseudogenized *T2r1* has ω2,others have ω1 (C) | ω1=0.69921 ω2=1.28800 | 3217.20 | 21 | A vs. C | 4.84 | 0.03 |
| The branches with pseudogenized *T2r1* has ω2=1,others have ω1 (D) | ω1=0.69816 ω2=1.00000 | 3217.68 | 20 | D vs. C | 0.96 | 0.33 |
| Each branch has its own ω(E) | Variable ω by branch | 3213.11 | 37 | C vs. E | 8.18 | 0.94 |

Tas2r2

| Models | ω | -lnL | np | Models compared | 2Δ (ln L) | p-value |
| --- | --- | --- | --- | --- | --- | --- |
| All branches have one ω(A) | 0.77950 | 2396.66 | 20 |  |  |  |
| All branches have one ω=1(B) | 1 | 2398.10 | 19 | B vs. A | 2.87 | 0.09 |
| The branches with pseudogenized *T2r2* has ω2,others have ω1 (C) | ω1=0.73811 ω2=1.01754 | 2396.33 | 21 | A vs. C | 0.67 | 0.41 |
| The branches with pseudogenized *T2r2* has ω2=1,others have ω1 (D) | ω1=0.73815 ω2=1.00000 | 2396.33 | 20 | D vs. C | 0.00 | 0.96 |
| Each branch has its own ω(E) | Variable ω by branch | 2388.37 | 37 | C vs. E | 15.92 | 0.46 |

***Tas2r3***

| Models | ω | -lnL | np | Models compared | 2Δ (ln L) | p-value |
| --- | --- | --- | --- | --- | --- | --- |
| All branches have one ω(A) | 0.84613 | 3410.35 | 20 |  |  |  |
| All branches have one ω=1(B) | 1 | 3411.39 | 19 | B vs. A | 2.08 | 0.15 |
| The branches with pseudogenized *T2r3* has ω2,others have ω1(C) | ω1= 0.78745 ω2= 0.95066 | 3410.03 | 21 | A vs. C | 0.63 | 0.43 |
| The branches with pseudogenized *T2r3* has ω2=1,others have ω1 (D) | ω1= 0.78735 ω2=1.00000 | 3410.07 | 20 | D vs. C | 0.07 | 0.79 |
| Each branch has its own ω(E) | Variable ω by branch | 3407.65 | 37 | C vs. E | 4.77 | 0.99 |

Tas2r5

| Models | ω | -lnL | np | Models compared | 2Δ (ln L) | p-value |
| --- | --- | --- | --- | --- | --- | --- |
| All branchs have one ω(A) | 0.83747 | 2327.14 | 16 |  |  |  |
| All branches have one ω=1(B) | 1 | 2327.87 | 15 | B vs. A | 1.46 | 0.23 |
| The branches with pseudogenized *T2r5* has ω2,others have ω1 (C) | ω1= 0.73026 ω2= 1.392901 | 2325.58 | 17 | A vs. C | 1.62 | 0.08 |
| The branches with pseudogenized *T2r5* has ω2=1,others have ω1 (D) | ω1= 0.73116 ω2=1.00000 | 2326.08 | 16 | D vs. C | 1.15 | 0.31 |
| Each branch has its own ω(E) | Variable ω1 by branch | 2321.94 | 29 | C vs. E | 8.78 | 0.84 |

Tas2r16

| Models | ω | -lnL | np | Models compared | 2Δ (ln L) | p-value |
| --- | --- | --- | --- | --- | --- | --- |
| All branchs have one ω(A) | 0.9358 | 2136.26 | 16 |  |  |  |
| All branches have one ω=1(B) | 1 | 2136.34 | 15 | B vs. A | 0.16 | 0.69 |
| The branches with pseudogenized *T2r16* has ω2,others have ω1 (C) | ω1= 0.89726 ω2= 1.03791 | 2136.18 | 17 | A vs. C | 0.16 | 0.67 |
| The branches with pseudogenized *T2r16* has ω2=1,others have ω1 (D) | ω1= 0.89731 ω2=1.00000 | 2136.19 | 16 | D vs. C | 0.01 | 0.91 |
| Each branch has its own ω (E) | Variable ωby branch | 2132.99 | 29 | C vs. E | 6.38 | 0.90 |

Tas2r39

| Models | ω | -lnL | np | Models compared | 2Δ (ln L) | p-value |
| --- | --- | --- | --- | --- | --- | --- |
| All branchs have one ω(A) | 0.73410 | 2779.58 | 16 |  |  |  |
| All branches have one ω=1(B) | 1 | 2782.34 | 15 | B vs. A | 5.51 | 0.02 |
| The branches with pseudogenized *T2r39* has ω2,others have ω1 (C) | ω1= 0.64399 ω2= 1.04450 | 2778.21 | 17 | A vs. C | 2.74 | 0.10 |
| The branches with pseudogenized *T2r39* has ω2=1,others have ω1 (D) | ω1= 0.64386 ω2=1.00000 | 2778.22 | 16 | D vs. C | 0.03 | 0.87 |
| Each branch has its own ω(E) | Variable ωby branch | 2774.49 | 29 | C vs. E | 7.43 | 0.83 |

Tas2r60

| Models | ω | -lnL | np | Models compared | 2Δ (ln L) | p-value |
| --- | --- | --- | --- | --- | --- | --- |
| All branchs have one ω(A) | 1.22021 | 2345.47 | 20 |  |  |  |
| All branches have one ω=1(B) | 1 | 2346.34 | 19 | B vs. A | 1.74 | 0.19 |
| The branches with pseudogenized *T2r60* has ω2,others have ω1(C) | ω1=0.89230  ω2= 1.98941 | 2342.45 | 21 | A vs. C | 6.04 | 0.01 |
| The branches with pseudogenized *T2r60* has ω2=1,others have ω1 (D) | ω1= 0.90478 ω2=1.00000 | 2346.21 | 20 | D vs. C | 7.52 | 0.01 |
| Each branch has its own ω(E) | Variable ω by branch | 2331.35 | 37 | C vs. E | 22.21 | 0.14 |

Under the assumption of a uniform ω across the tree, we got an average ω approximate to 1 ranging from 0.73410 to 1.22021 for all the seven *Tas2rs* analyzed in model A, in which model A in *Tas2r1* and *Tas2r39* are better than model B which assumed a fixed ω=1 across the tree (*p*=0.02; *p*=0.02), indicating that functional constraints were almost completely removed, while for the left five *Tas2rs* model B was better, indicative of completely functional relaxation. To analyze selective pressure on branches with a pseudogenised *Tas2r* , we performed a two-ratio model in which branches with a pseudogenised *Tas2r* had a ω2 which was proved to near to 1 for seven *Tas2rs* , other branches had a ω1 (model C). In comparison with model A, we found model C was not better excluding *Tas2r1* and *Tas2r60* (*p*=0.03; *p*=0.01). Further, model D which assumed a fixed ω2 =1 in pseudogenised branches and a ω1 in other branches was better than model C for almost all Tas2rs excluding Tas2r60, suggesting that selective pressure was already completely removed from almost *Tas2rs*. Finally, model E which allows each branch has a ω was not better for all *Tas2rs*, suggesting ω was not variable across the tree.
