# Supplementary material for: The loss of taste genes in cetaceans
Source: BMC Evol Biol. 2014 Oct 12;14:218. doi: 10.1186/s12862-014-0218-8 (PMC4232718; doi:10.1186/s12862-014-0218-8)
Supplement: Additional file 7: Table S16. — Physicochemical properties under positive destabilizing selection in ENaCα, β, γ. [file 12862_2014_218_MOESM7_ESM.doc]

**Table S16: Physicochemical properties under positive destabilizing selection in ENaC α, β, γ**.

| Amino acid properties | Category | z-score |
| --- | --- | --- |
| ENaC α |  |  |
| Alpha-helical tendencies | 6 | 6.904 |
| Alpha-helical tendencies | 8 | 2.985 |
| Bulkiness | 6 | 1.72 |
| Coil tendencies | 6 | 1.649 |
| Coil tendencies | 8 | 2.433 |
| Compressibility | 7 | 2.589 |
| Compressibility | 8 | 2.555 |
| Equilibrium constant (ionization of COOH) | 8 | 2.329 |
| Isoelectric point | 8 | 2.125 |
| Long-range non-bonded energy | 8 | 1.961 |
| Power to be at the C-terminal | 6 | 2.181 |
| Power to be at the N-terminal | 6 | 3.602 |
| Power to be at the N-terminal | 8 | 2.657 |
| Surrounding hydrophobicity | 6 | 2.951 |
| Turn tendencies | 7 | 4.404 |
| ENaC β |  |  |
| Alpha-helical tendencies | 6 | 3.995 |
| Equilibrium constant (ionization of COOH) | 8 | 3.357 |
| Isoelectric point | 7 | 3.331 |
| Power to be at the C-terminal | 6 | 4.272 |
| Power to be at the middle of alpha-helix | 7 | 2.756 |
| ENaC γ |  |  |
| Alpha-helical tendencies | 6 | 1.992 |
| Equilibrium constant (ionization of COOH) | 8 | 5.278 |
| Isoelectric point | 7 | 4.77 |
| Power to be at the C-terminal | 6 | 3.465 |
