# Supplementary material for: The loss of taste genes in cetaceans
Source: BMC Evol Biol. 2014 Oct 12;14:218. doi: 10.1186/s12862-014-0218-8 (PMC4232718; doi:10.1186/s12862-014-0218-8)
Supplement: Additional file 8: Table S17. — PCR primers for each taste receptor gene. [file 12862_2014_218_MOESM8_ESM.doc]

**Table S17**. PCR primers for each taste receptor gene.

| Gene | Exons | Primer names | Primer sequences | |
| --- | --- | --- | --- | --- |
| *Tas1r1* | Exon 1 | T1-1 F | GCAGTGCAAGGACAGAACCCACAAC | |
|  |  | T1-1 R | AGGACTCTAACAGCCAGGGCAAAAG | |
|  | Exon 2 | Tas1re F | CACCCTGACTCATATAGCAGGTTTC | |
|  |  | Tas1re R | AGAAGGGCAGAAGTTTCCG | |
|  | Exon 3 | T1-3-1 F | ACTGGGCCATCTCCAGACACATCAG | |
|  |  | T1-3-1 R | CTGAAACAAACAGGGATAACGGCGC | |
|  |  | T1-3-4 F | GCCATCTCCAGACACATC | |
|  |  | T1-3-4 R | GACTAACACGAGCCTCAG | |
|  | Exon 4 | T1-4-2 F | ACTCAGGAGGCTGGATAG | |
|  |  | T1-4-2 R | GGACACTCGTTCTTAGGAT | |
|  | Exon 5 | T1R1-5 F | CAGATGCCCAGAGATCTAGTTTTCC | |
|  |  | T1R1-5 R | GGTCCTTTCATCTGCTCTACAGACC | |
|  |  | T1-5 F | GAAGGCAGATGCCCAGAGATCTAGT | |
|  |  | T1-5 R | CTAAGACCCAATGCAGCCAAAAACG | |
|  | Exon 6 | T1-6-q F | GCTCACATTGGATCATAGCCTCAAG | |
|  |  | T1-6-q R | GTTGTTGAGCTGATCACCACCAACG | |
|  |  | T1-6-h F | CGATCCGCTCCTTCCAACTTGTGTT | |
|  |  | T1-6-h R | CTGAGCTCTCCCGTGGGCTTACACT | |
| *Tas1r2* | Exon 1 | T1R2 1 F3 | GAATCAGTGGCTGCTGTTGTGGAAC | |
|  |  | T1R2 1 R3 | ATTTTAATAACGCGGACTGTGCCGG | |
|  | Exon 2 | T1R2 2 F2 | GATGGAGGCTATGGTGGGTAATGGT | |
|  |  | T1R2 2 R2 | TTAGAGATGGGGAAACTGAGGCTTG | |
|  | Exon 3 | T1R2 3 F1 | GAGGATGGTGAGGAGGAA | |
|  |  | T1R2 3 R1 | CGTGAGGATGGTGTTGAA | |
|  |  | T1R2 3 F2 | TCGCTAATACCTGCTGGA | |
|  |  | T1R2 3 R2 | CTAGGTGATGCCGAACTG | |
|  | Exon 4 | T1R2 4 F1 | TAAGAAGGTGCCAACAGAG | |
|  |  | T1R2 4 R1 | AAGCCAGGAAGTTATAGAGG | |
|  | Exon 5 | T1R2 5 F2 | TGGATGTAGATGCTGAGAAG | |
|  |  | T1R2 5 R2 | GACGGAGGAGGTGAAGTA | |
|  | Exon 6 | T1R2 6 F1 | CTGTGTCTTCGTGGCATT | |
|  |  | T1R2 6 R1 | TAATCGTTGAGGTGGCTTC | |
|  | Exon 1 | PKD2L1 1 F1 | CTCCCTTTCCTTTGCCCTCT | |
| *Pkd2l1* | Exon 1 | PKD2L1 1 R1 | GGGCAGTCTAAGGCATCATC | |
|  | Exon 2 | PKD2L1 2 F1 | ATCTGTACCATCTCCAGTG | |
|  |  | PKD2L1 2 R1 | AAGTTCCTGGTTCTCAGC | |
|  | Exon 3-4 | pkd2l1 3-4 F1 | AACCGTCAGCCTCCTTAG | |
|  |  | pkd2l1 3-4 R1 | GACTCACTCACGCTATGC | |
|  | Exon 5 | PKD2L1 5 F1 | AACGGGAGATGTGTGGAT | |
|  |  | PKD2L1 5 R1 | CAGCCAATGATAAAGAAGTC | |
|  | Exon 6 | PKD2L1 6 F1 | CAGTCTACAACGCCAACAT | |
|  |  | PKD2L1 6 R1 | GAGAAATGGAGCCAAGCAAT | |
|  | Exon 7-8 | PKD2L1 7-8 F1 | CATCTTCGTCACCTTCCTAT | |
|  |  | PKD2L1 7-8 R1 | TTCCTACCGCTCTTCAGT | |
|  | Exon 8-9 | PKD2L1 8-9 F1 | CGCTGTCAACCTCTTCTT | |
|  |  | PKD2L1 8-9 R1 | TCCTTCTCCTTCCATCCTT | |
|  | Exon 10 | PKD2L1 10 F1 | GCTTCTCCGCCTATTCTC | |
|  |  | PKD2L1 10 R1 | CCTCCTCTTCATCTCTTACTC | |
|  | Exon 11 | PKD2L1 11 F1 | GGCGTGGGAGTAAGAGATGA | |
|  |  | PKD2L1 11R1 | GTGACCTTAGCCCATTTCT | |
|  | Exon 12 | PKD2L1 12 F1 | GGCAGGTCAGAAGTCATT | |
|  |  | PKD2L1 12 R1 | GCTCAGAAGGAATTGTCAG | |
|  | Exon 13 | PKD2L1 13 F1 | CAACTCTGATCCTTAGCTG | |
|  |  | PKD2L1 13 R1 | GATGGTAGAGATCAGCTGT | |
|  | Exon 15 | PKD2L1 15 F1 | TCCTGGGGCAACATGTTC | |
|  |  | PKD2L1 15 R1 | ACCTGCTGCCCTAGAAGTG | |
|  | Exon 16 | PKD2L1 16 F1 | GATCTACTGCCAGTACCCA | |
|  |  | PKD2L1 16 R1 | ATCACCAGGCAAACCCAC | |
| *T2rs* | | | | |
| *T2r1* |  | T2R1-2 R2 | | AAATGTCTCAGGTTGGAC |
|  |  | T2R1-2 F3 | | GGTGAGGATATCCAAGTTG |
|  |  | T2R1 F3 | | TCATGGTAGAGATGAAGGAT |
|  |  | T2R1 R3 | | AGGGTACTGACATTGATGT |
| *T2r2* |  | T2R2-1 F2 | | GTCAGCTCGTCTTCATGT |
|  |  | T2R2-1 R2 | | CCTCTTTAGCATGGCATC |
|  |  | T2R2-1 F3 | | CCAGAAATTGGCAGAAGGTC |
|  |  | T2R2-1 R3 | | TCACAGATATGGCCAGAGG |
|  |  | T2R2-2 F1 | | GCTGAAATTCAGGATCTC |
|  |  | T2R2-2 R1 | | TTAAGTACACAAGTCCTGG |
| *T2r3* |  | T2R3 1 F2 | | GTAACACTGGACACTACTC |
|  |  | T2R3 1 R2 | | GACGGCACTGCTACATGAT |
|  |  | T2R3 1 F3 | | TTCCATTCTGGGTGGCTCAG |
|  |  | T2R3 1 R3 | | CCATGAACATCTGCTTCAGC |
|  |  | T2R3 2 F1 | | ACCATCTGAGCATTTGGC |
|  |  | T2R3 2 R1 | | CAGGTCTAAGGACTCCTAGG |
| *T2r5* |  | T2R5 F1 | | GGTATCATGACCCTTGAG |
|  |  | T2R5 R1 | | GGACTTCAGGACAGTGAT |
| *T2r16* |  | T2R16 1 F1 | | TCAAATCACCTCAGGAGC |
|  |  | T2R16 1 R1 | | TTAGGGCTGCTCAGCAT |
|  |  | T2R16 2 F1 | | GGTACTTCAGTATCGTCTGG |
|  |  | T2R16 2 R1 | | CAGTCATGGAATGGGTC |
|  |  | T2R16 2 F2 | | TCATTCTGGTTGACCAGC |
|  |  | T2R16 2 R2 | | TCAAGAGTCCTGCAGGTTAG |
| *T2r38* |  | T2R38 F1 | | TGGTGACTCTGACTGCCAC |
|  |  | T2R38 R1 | | TCTGGCATCCTGGGATCTG |
| *T2r39* |  | T2R39 1 F1 | | ACGCAGTTATAGGGTGACT |
|  |  | T2R39 1 R1 | | GGATGAACGTGATCAGAGG |
|  |  | T2R39 1 F2 | | GAGCGTCTACTCAAGTTCC |
|  |  | T2R39 1 R2 | | TCACTAAGACTGTGCCCATG |
|  |  | T2R39 2 F2 | | TCCTGGCTTCACTGATCTG |
|  |  | T2R39 2 R2 | | GTGAAGATGGCGGATTTC |
| *T2r60* |  | T2R60 F1 | | GGTCTAAGCTCCCATGTAAG |
|  |  | T2R60 R1 | | AGTCTCTGAGGCTCTTCATC |
| *T2r62a* |  | T2R62A 1 F1 | | CCATGTTCCTACATCTGGC |
|  |  | T2R62A 1 R1 | | CTATGGTCCCTCATCTGC |
|  |  | T2R62A 2 F1 | | CTTGCAGGCGACATGATTG |
|  |  | T2R62A 2 R1 | | GCTGCTTACGTAGAGAGTG |
|  |  | T2R62A 2 F2 | | CCCTAGGACTTCATCAACT |
|  |  | T2R62A 2 R2 | | GGTTGACCACAGAACAACTG |
| *T2r62b* |  | T2R62B F2 | | ACACTCTCACTTTCTGGC |
|  |  | T2R62B R2 | | CCTTGTGCATTCTTGCCAC |
|  |  | T2R62B F3 | | GTCTCCCRGTTCTGTCTG |
|  |  | T2R62B R3 | | CTCGCCGAAGCCTCTTC |
| *Scnn1a* | Exon 1 | SCNN1A 1 F1 | | CTCAACCTCTACAGCCTAC |
|  |  | SCNN1A 1 R1 | | AACCTCCGTCAGATTCATC |
|  |  | SCNN1A 1 F2 | | AACCACACCACCATCCAT |
|  |  | SCNN1A 1 R2 | | CCAGAACCTCCGTCAGAT |
|  | Exon 2 | SCNN1A 2 F1 | | CCGACCTCTCCGGTTACAG |
|  |  | SCNN1A 2 R1 | | CAAGCTGGAGCCCAGAGAG |
|  | Exon 3 | SCNN1A 3 F1 | | CTCTGGTCTCTGTGTCTAGTG |
|  |  | SCNN1A 3 R1 | | AGTGCTCTCAACTCGTCAT |
|  | Exon 4-5 | SCNN1A 4 F1 | | CCGTGAAGTAGGGATGAT |
|  |  | SCNN1A 4 R1 | | GACTGGGACAGAATTCCT |
|  |  | SCNN1A 5 F1 | | ACAAGAACAACTCCAACCT |
|  |  | SCNN1A 5 R1 | | ACCTTACCTCCTCTCCTATC |
|  |  | SCNN1A 45 F3 | | CTCATCCTTGCTGCTTGT |
|  |  | SCNN1A 45 R3 | | ACCTTACCTCCTCTCCTATC |
|  | Exon 6-7 | SCNN1A 67 F1 | | TGACCTCTGACTTCTCCAT |
|  |  | SCNN1A 67 R1 | | ATTCCTCCGTAAGCCATTC |
|  | Exon 8-9 | SCNN1A 89 F1 | | CTCGCTGTGTCTCATTATTC |
|  |  | SCNN1A 89 R1 | | CCTCCAATCAACCGTATCA |
|  | Exon 10-11 | SCNN1A 1011 F2 | | TAAGCTCCAGGACGCCTTC |
|  |  | SCNN1A 1011 R2 | | CATCTCCACCACAGACAGCA |
|  | Exon 12 | SCNN1A 12-F3 | | AGGGCAAGGGTGAGGATTAGA |
|  |  | SCNN1A 12-R3 | | AGGACCAGCGGGTACA |
| *Scnn1g* | Exon 1 | SCNN1G 1 F1 | | GCAAATATCCTGGAGCAAG |
|  |  | SCNN1G 1 R1 | | TGACCCACTTAGAACAGGTC |
|  | Exon 2 | SCNN1G 2 F1 | | GGGCTGTCAAGGTAACAG |
|  |  | SCNN1G 2 R1 | | AACCGTGAGGATGTGCATC |
|  | Exon 3 | SCNN1G 3 F1 | | TCAGTTCCTTACAGTCCTTC |
|  |  | SCNN1G 3 R1 | | TCACCTCCATCTCCATCC |
|  | Exon 4 | SCNN1G 4 F1 | | AAGTCAAGTGTTGGCTGA |
|  |  | SCNN1G 4 R1 | | CACGTCTGGATTTAAGTCCAT |
|  | Exon 5 | SCNN1G 5 F1 | | AGTGATCAGATTCACCACTGG |
|  |  | SCNN1G 5 R1 | | CCTCACTGGTTATCCTTC |
|  |  | SCNN1G 5 F2 | | TGCTTAGCAAACCTGCAGT |
|  |  | SCNN1G 5 R2 | | ATCCWAAATGGCATCCTGTC |
|  | Exon 6 | SCNN1G 6 F1 | | AATAGCATCAAGCTCCTAG |
|  |  | SCNN1G 6 F1 | | CTCCTAGTAGGTGCTCAATA |
|  | Exon 7 | SCNN1G 7 F1 | | AGTCGGCTGTGACATGAG |
|  |  | SCNN1G 7 R1 | | CGCCTTAGCTATTGCCAG |
|  | Exon 8-11 | SCNN1G 8-11 F1 | | GAACAGGTAACTGAGGCTC |
|  |  | SCNN1G 8-11 R1 | | GGTGGAGAAAGAAGTGGC |
|  |  | SCNN1G 8-11 F2 | | GAACAGGTAACTGAGGCTC |
|  |  | SCNN1G 8-11 R2 | | TACTGTGGGTCATGGTCA |
|  |  | SCNN1G 11 F1 | | CCAGAAACCAGGTTCTAAG |
|  |  | SCNN1G 11 R1 | | TGGGCAGGTCATCGTCTAT |
|  | Exon 12 | SCNN1G 12 F1 | | GGACAGACTTGGCCAAAC |
|  |  | SCNN1G 12 R1 | | AACTCTGTCAGGCTTCAC |
| *Scnn1b* | Exon 3 | SCNN1B 3F1 | | GTTGGTTGGATGGATGGAT |
|  |  | SCNN1B 3R1 | | CTTGAAGCCGATGGAGAG |
|  | Exon 4 | SCNN1B 4F1 | | CCTACACAGCACACCTAC |
|  |  | SCNN1B 4R1 | | CCAGCCACATCTCAACTT |
|  |  | SCNN1B 4F2 | | GAGGAGCATTCTTCTCACAT |
|  |  | SCNN1B 4R1 | | CCAGCCACATCTCAACTT |
|  | Exon 5 | SCNN1B 5F1 | | CCACAGGAACCTCTCTTGC |
|  |  | SCNN1B 5R1 | | CACCTTGCCTGGGAAATTAG |
|  |  | SCNN1B 5F2 | | TGTGAGTCCTTGTGACTGCAG |
|  |  | SCNN1B 5R2 | | TTCCCTAAAGCGATTCCCTC |
|  | Exon 6 | SCNN1B 6F1 | | ACCTGTTGCTCACCTCTC |
|  |  | SCNN1B 6R1 | | CACTACTTCACCTGCTCTC |
|  |  | SCNN1B 6F2 | | CAGGTAAAGGGACAGAATG |
|  |  | SCNN1B 6R2 | | TATGTGAGCGTGGGCAAA |
|  |  | SCNN1B 6F3 | | TCTCTGAGCAGTAATCTACC |
|  |  | SCNN1B 6R3 | | GAGCACACCAGTCACTAC |
|  | Exon 7 | SCNN1B 7F1 | | GGAGAGCATATCGCAGAG |
|  |  | SCNN1B 7R1 | | GGAGTAGGTCGTGTTGTAG |
|  | Exon 8 | SCNN1B 8F1 | | TCTCCTCACCATCCACTC |
|  |  | SCNN1B 8R1 | | ACATACACAAGGCTTCTTCT |
|  | Exon 9 | SCNN1B 9F1 | | CCACTCAACCTAAGCACAG |
|  |  | SCNN1B 9R1 | | CCTCTCATCATCTGGTCTAC |
|  |  | SCNN1B 9F2 | | GGGTGGATGTAGGTGATT |
|  |  | SCNN1B 9R2 | | GTGGGTGGAAGTTAGCAG |
|  | Exon 12 | SCNN1B 12F4 | | ATCGCACCATTGAGGAGT |
|  |  | SCNN1B 12R4 | | TGCCCAGTCAGACAAAGAAC |
|  |  | SCNN1B 12F6 | | GTCCCTTAAGCAGTGTCAG |
|  |  | SCNN1B 12R6 | | CATCCGTACCAGCACCGTTA |
