# Supplementary material for: The loss of taste genes in cetaceans
Source: BMC Evol Biol. 2014 Oct 12;14:218. doi: 10.1186/s12862-014-0218-8 (PMC4232718; doi:10.1186/s12862-014-0218-8)
Supplement: Additional file 9: Table S18. — Accession numbers for species used in PAML analysis. [file 12862_2014_218_MOESM9_ESM.doc]

**Table S18. Accession numbers for species used in our PAML analysis.**

| Species | Gene | Accession number |
| --- | --- | --- |
| *Bos taurus* | *Tas1r1* | ENSBTAG00000016734 |
| *Canis lupus familiaris* |  | ENSCAFG00000019609 |
| *Pteropus vampyrus* |  | ENSLAFG00000030193 |
| *Equus caballus* |  | ENSECAG00000011699 |
| *Homo sapiens* |  | ENSG00000173662 |
| *Mus musculus* |  | ENSMUSG00000028950 |
| *Sus scrofa* |  | ENSSSCG00000003382 |
| *Ailuropoda melanoleuca* |  | HM468452 |
| *Bos taurus* |  | ENSBTAG00000015246 |
| *Ailuropoda melanoleuca* |  | ENSAMEG00000017108 |
| *Homo sapiens* |  | ENSG00000179002 |
| *Mus musculus* |  | [ENSMUSG00000028738](http://asia.ensembl.org/Mus_musculus/Gene/Summary?g=ENSMUSG00000028738) |
| *Pteropus vampyrus* |  | ENSPVAG00000003398 |
| *Zalophus californianus californianus* | *Tas1r2* | JN130349 |
| *Arctocephalus forsteri* |  | JN130350 |
| *Phoca vitulina richardii* |  | JN130351 |
| *Amblonyx cinereus* |  | JN130352 |
| *Bos taurus* |  | ENSBTAG00000010742 |
| *Equus caballus* |  | ENSCAFG00000009644 |
| *Canis lupus familiaris* | *pkd2l1* | ENSECAG00000023606 |
| *Ailuropoda melanoleuca* |  | ENSAMEG00000006758 |
| *Pteropus vampyrus* |  | ENSPVAG00000008030 |
| *Homo sapiens* |  | ENSG00000107593 |
| *Bos taurus* |  | ENSBTAG00000002631 |
| *Equus caballus* |  | ENSECAG00000001443 |
| *Canis lupus familiaris* |  | ENSCAFG00000015196 |
| *Myotis lucifugus* | *Scnn1a* | ENSMLUG00000014284 |
| *Ailuropoda melanoleuca* |  | ENSAMEG00000014498 |
| *Homo sapiens* |  | ENSG00000111319 |
| *Mus musculus* |  | ENSMUSG00000030340 |
| *Bos taurus* |  | ENSBTAG00000010163 |
| *Sus scrofa* |  | ENSSSCG00000007836 |
| *Equus caballus* |  | ENSECAG00000021259 |
| *Pteropus vampyrus* | *Scnn1g* | ENSPVAG00000011219 |
| *Canis lupus familiaris* |  | ENSCAFG00000017690 |
| *Ailuropoda melanoleuca* |  | ENSAMEG00000011313 |
| *Homo sapiens* |  | ENSG00000166828 |
| *Mus musculus* |  | ENSMUSG00000000216 |
| *Bos taurus* |  | [ENSBTAG00000012290](http://asia.ensembl.org/Bos_taurus/Gene/Summary?g=ENSBTAG00000012290) |
| *Equus caballus* |  | [ENSECAG00000014993](http://asia.ensembl.org/Equus_caballus/Gene/Summary?g=ENSECAG00000014993) |
| *Canis lupus familiaris* | *Scnn1b* | [ENSCAFG00000017687](http://asia.ensembl.org/Canis_familiaris/Gene/Summary?g=ENSCAFG00000017687) |
| *Pteropus vampyrus* |  | [ENSPVAG00000002693](http://asia.ensembl.org/Pteropus_vampyrus/Gene/Summary?g=ENSPVAG00000002693) |
| *Homo sapiens* |  | [ENSG00000168447](http://asia.ensembl.org/Homo_sapiens/Gene/Summary?g=ENSG00000168447) |
| *Tursiops truncatus* |  | [ENSTTRG00000005185](http://asia.ensembl.org/Tursiops_truncatus/Gene/Summary?g=ENSTTRG00000005185) |
| *Sus scrofa* |  | [ENSSSCG00000028923](http://asia.ensembl.org/Sus_scrofa/Gene/Summary?g=ENSSSCG00000028923) |
| *Bota -T2R1* |  | [AB249703](http://www.ebi.ac.uk/ebisearch/redirect.ebi?url=%2Fena%2Fdata%2Fview%2FAB249703&digest=B0B6B4) |
| *Cafa-T2R1* |  | [AB249684](http://www.ebi.ac.uk/ebisearch/redirect.ebi?url=%2Fena%2Fdata%2Fview%2FAB249684&digest=AF65B8) |
| *Bota-T2R2p* |  | [AB249704](http://www.ebi.ac.uk/ebisearch/redirect.ebi?url=%2Fena%2Fdata%2Fview%2FAB249704&digest=57EE38) |
| *Cafa-T2R2* |  | [AB249685](http://www.ebi.ac.uk/ebisearch/redirect.ebi?url=%2Fena%2Fdata%2Fview%2FAB249685&digest=D8BDF8) |
| *Cafa-T2R3* |  | [AB249686](http://www.ebi.ac.uk/ebisearch/redirect.ebi?url=%2Fena%2Fdata%2Fview%2FAB249686&digest=569FCB) |
| *Bota-T2R3* |  | [AB249705](http://www.ebi.ac.uk/ebisearch/redirect.ebi?url=%2Fena%2Fdata%2Fview%2FAB249705&digest=CD796C) |
| *Cafa-T2R5* | *Tas2rs* | [AB249688](http://www.ebi.ac.uk/ebisearch/redirect.ebi?url=%2Fena%2Fdata%2Fview%2FAB249688&digest=F1118B) |
| *Bota-T2R5p* |  | AB249707 |
| *Bota-T2R16* |  | [AB249716](http://www.ebi.ac.uk/ebisearch/redirect.ebi?url=%2Fena%2Fdata%2Fview%2FAB249716&digest=750FD0) |
| *Cafa-T2R38* |  | [AB249694](http://www.ebi.ac.uk/ebisearch/redirect.ebi?url=%2Fena%2Fdata%2Fview%2FAB249694&digest=49E173) |
| *Cafa-T2R39* |  | [AB249695](http://www.ebi.ac.uk/ebisearch/redirect.ebi?url=%2Fena%2Fdata%2Fview%2FAB249695&digest=D8841E) |
| *Bota-T2R39* |  | [AB249717](http://www.ebi.ac.uk/ebisearch/redirect.ebi?url=%2Fena%2Fdata%2Fview%2FAB249717&digest=CA473F) |
| *Bota-T2R56* |  | AB249725 |
| *Bota-Tas2r60* |  | xm_002687121 |
| *Bota-T2R62p* |  | [AB249726](http://www.ebi.ac.uk/ebisearch/redirect.ebi?url=%2Fena%2Fdata%2Fview%2FAB249726&digest=E26725) |
| *Cafa-T2R62p* |  | [AB249701](http://www.ebi.ac.uk/ebisearch/redirect.ebi?url=%2Fena%2Fdata%2Fview%2FAB249701&digest=DCE394) |
|  |  |  |
